# Fluorinated Zinc and Copper Phthalocyanines as Efficient Third Components in Ternary Bulk Heterojunction Solar Cells

**DOI:** 10.1021/acsaem.1c00734

**Published:** 2021-05-12

**Authors:** Alfonsina Abat Amelenan Torimtubun, Jorge Follana-Berná, José G. Sánchez, Josep Pallarès, Ángela Sastre-Santos, Lluis F. Marsal

**Affiliations:** † Department of Electric, Electronic and Automatic Engineering, Universitat Rovira i Virgili, Av. Països Catalans 26, Tarragona 43007, Spain; ‡ Área de Química Orgánica, Instituto de Bioingeniería, Universidad Miguel Hernández de Elche, Av. de la Universidad s/n, Elche 03202, Spain

**Keywords:** fluorinated phthalocyanines, third component, ternary organic solar cells, bulk heterojunction, non-fullerene acceptors

## Abstract

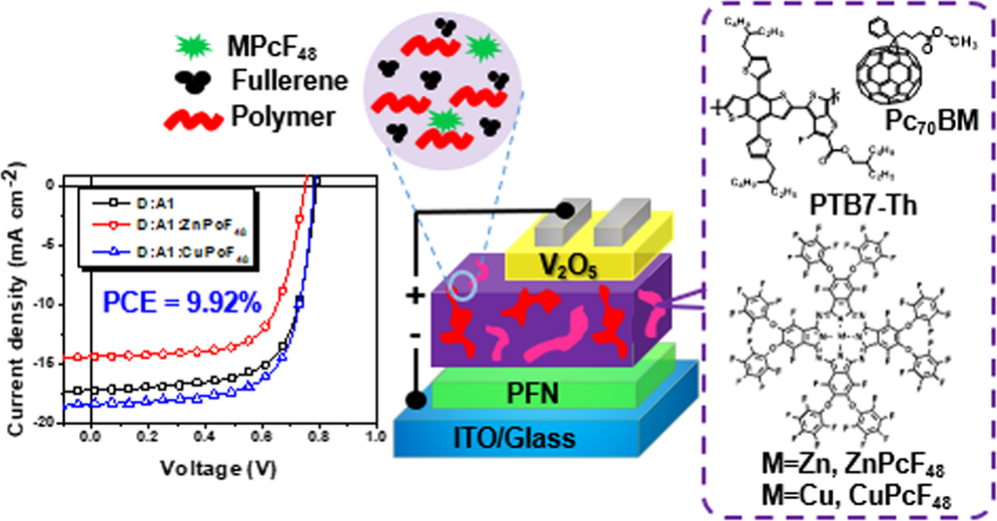

Fluorinated zinc and copper metallophthalocyanines MPcF_48_ are synthesized and incorporated as third component small molecules in ternary organic solar cells (TOSCs). To enable the high performance of TOSCs, maximizing short-circuit current density (*J*_SC_) is crucial. Ternary bulk heterojunction blends, consisting of a polymer donor PTB7-Th, fullerene acceptors PC_70_BM, and a third component MPcF_48_, are formulated to fabricate TOSCs with a device architecture of ITO/PFN/active layer/V_2_O_5_/Ag. Employing copper as metal atom substitution in the third component of TOSCs enhances *J*_SC_ as a result of complementary absorption spectra in the near-infrared region. In combination with *J*_SC_ enhancement, suppressed charge recombination, improved exciton dissociation and charge carrier collection efficiency, and better morphology lead to a slightly improved fill factor (FF), resulting in a 7% enhancement of PCE than those of binary OSCs. In addition to the increased PCE, the photostability of TOSCs has also been improved by the appropriate addition of CuPcF_48_. Detailed studies imply that metal atom substitution in phthalocyanines is an effective way to improve *J*_SC_, FF, and thus the performance and photostability of TOSCs.

## 1 Introduction

Bulk heterojunction organic solar cells (OSCs) featuring properties of low cost, light-weight, solution-processed, large area, semitransparent, and flexible substrates have attracted great interest for decades.^1−6^ OSCs are typically composed of an active layer consisting of electron donor (D) and electron acceptor (A) materials in the blends of single-junction OSCs or tandem cells.^7−10^ However, due to low charge carrier mobility, limited spectral absorption, and high thermal loss in organic materials, the tandem cell strategy is preferred as an effective way to address these issues.^11,12^ Tandem cells can suppress the thickness constraint due to the low mobility of organic materials in a single-junction cell, providing a wide and efficient optical absorption spectrum.^13^ Meanwhile, thermalization losses in a single-junction cell can also be avoided thanks to the tunability band structure of the active organic materials in tandem cells.^14^ Nevertheless, the complex fabrication process and high upfront cost limit the practical application of tandem cells.^15^ In recent years, ternary organic solar cells (TOSCs) have been proved to be one of the most promising methods to improve device performance by adding a third component into a binary system, resulting in a single three-component photoactive layer.^16−18^ TOSCs could combine each merit of binary and tandem solar cells to extend light absorption, simplify device fabrication, and increase device efficiency in a single-junction device.^16,19−21^

In a ternary blend, the addition of a third component (secondary donor D_2_ or secondary acceptor A_2_) provides broadened light harvesting, optimizes the film morphology, and facilitates better charge transport and exciton dissociation.^22−24^ The dominant fullerene derivatives have been widely used in OSCs as a primary electron acceptor (A_1_) or A_2_. However, fullerene acceptors show weak light absorption, difficult energetic tunability, and thermal instability.^21,25^ Recently, non-fullerene acceptors (NFAs) have been synthesized to overcome the disadvantages of FAs. The rapid progress of highly efficient NFA materials has offered a new opportunity for studying ternary OSCs due to the great tunability of chemical structures, optical and electronic properties of NFAs, and their ability to phase separate into nanoscopic domains in blended thin films.^26−31^

**Figure 1:**
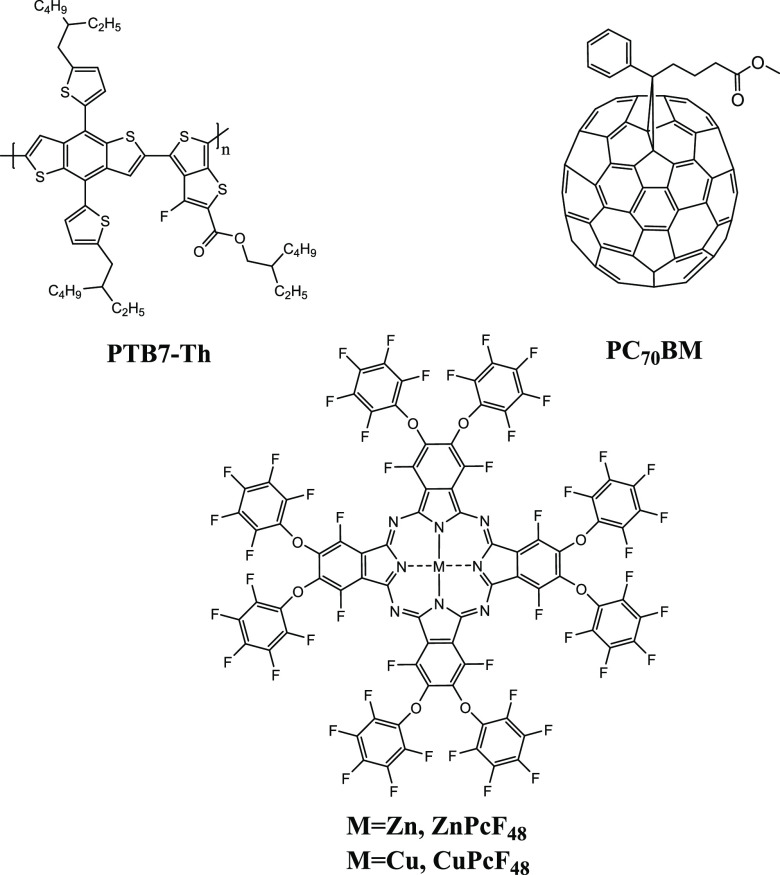
Chemical structures of PTB7-Th, PC_70_BM, ZnPcF_48_, and CuPcF_48_ in ternary bulk heterojunction solar cells.

**Scheme 1:**
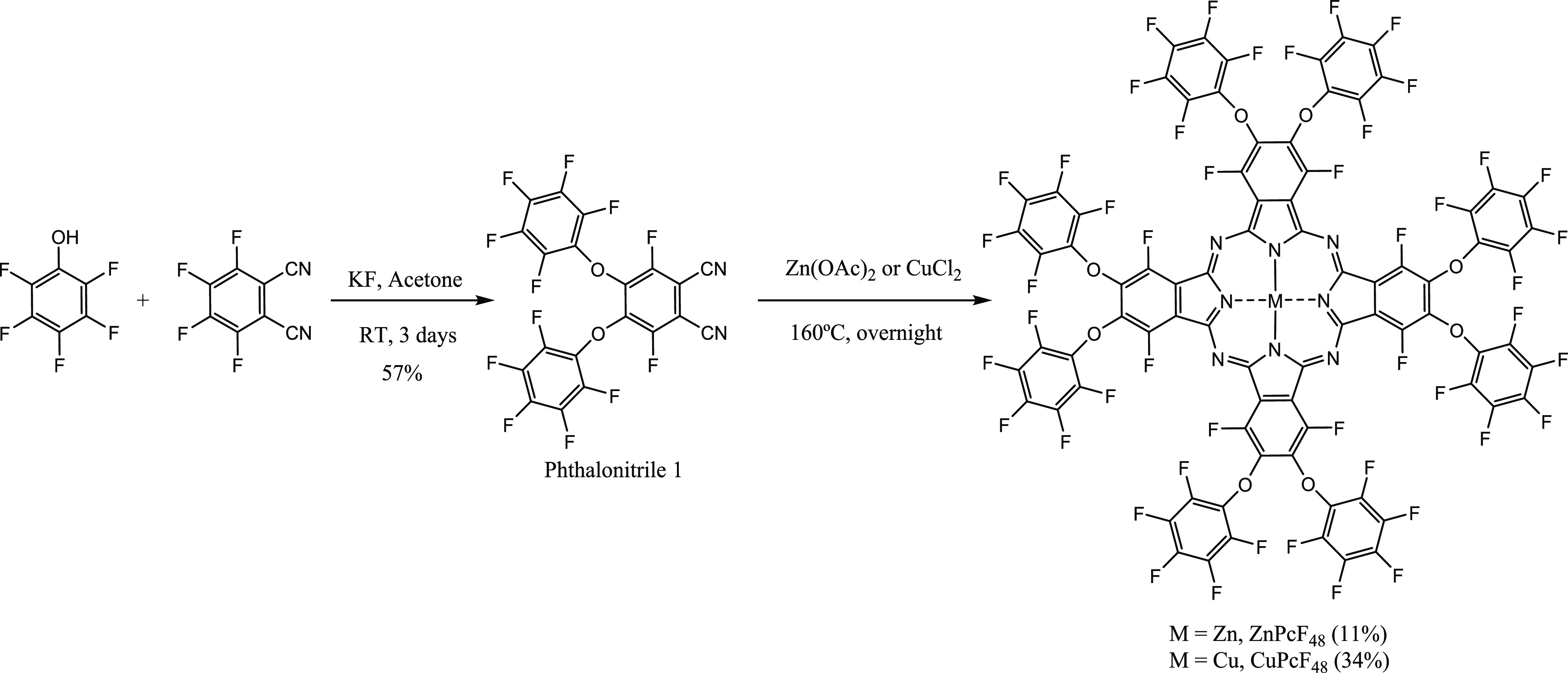
Synthetic Route of ZnPcF_48_ and CuPcF_48_

Different small molecules from phthalocyanine (Pc) derivatives have been described to provide alternative photovoltaic semiconductor materials for OSC applications.^32−35^ Pcs play an important role as NFAs due to their high molar extinction coefficients, stability, and the highest occupied molecular orbital (HOMO)/lowest unoccupied molecular orbital (LUMO) energy levels complementary with the energy levels of the other two components.^36,37^ However, Pcs have poor solubility in organic solvents and most of the Pcs used to date in ternary solar cells have been SiPcs thanks to the functionalization of the axial positions that avoid aggregation processes.^38−42^ Due to this problem, to date, not too many Pcs have been incorporated in ternary solar cells. Different studies have been performed using CuPc^43^ and ZnPc^44^ as third components and in all of the cases resulted in an efficiency improvement compared to its two-component counterpart. In all these studies, Pcs act as an efficient transporter between a fullerene derivative and a polymer. For this reason, a deeper study should be done studying the influence of the substituents and the metal of the Pcs on the PV performance. In this work, we have prepared two new zinc and copper phthalocyanines substituted with electronegative fluorine atoms in benzene rings to enhance their solubility and their electron acceptor capability. Hence, ZnPcF_48_ and CuPcF_48_ (Scheme 1) have been synthesized and incorporated into the ternary system based on PTB7-Th:PC70BM (Figure 1) to understand their influence on the OSC device performance.

## 2 Results and Discussion

### 2.1 Synthesis and Materials Properties

The synthesis of ZnPcF_48_ and CuPcF_48_ was accomplished by statistical cyclotetramerization of 3,6-difluoro-4,5-bis(pentafluorophenoxy)phthalonitrile **1** using Zn(OAc)_2_ or CuCl_2_ as a template, respectively (Scheme 1). Phthalonitrile **1** was synthesized by nucleophilic aromatic substitution of pentafluorophenol to tetrafluorophthalonitrile and characterized by FT-IR, ^13^C NMR, and MALDI-TOF-MS. The ^13^C NMR spectrum was recorded without fluoride decoupling and showed several multiplets due to the coupling of ^13^C with ^19^F. The ^13^C signal assignments of phthalonitrile **1** were performed by a comparative study between pentafluorophenol and tetrafluorophthalonitrile ^13^C NMR spectra (see Figures S1 and S2).

**Figure 2:**
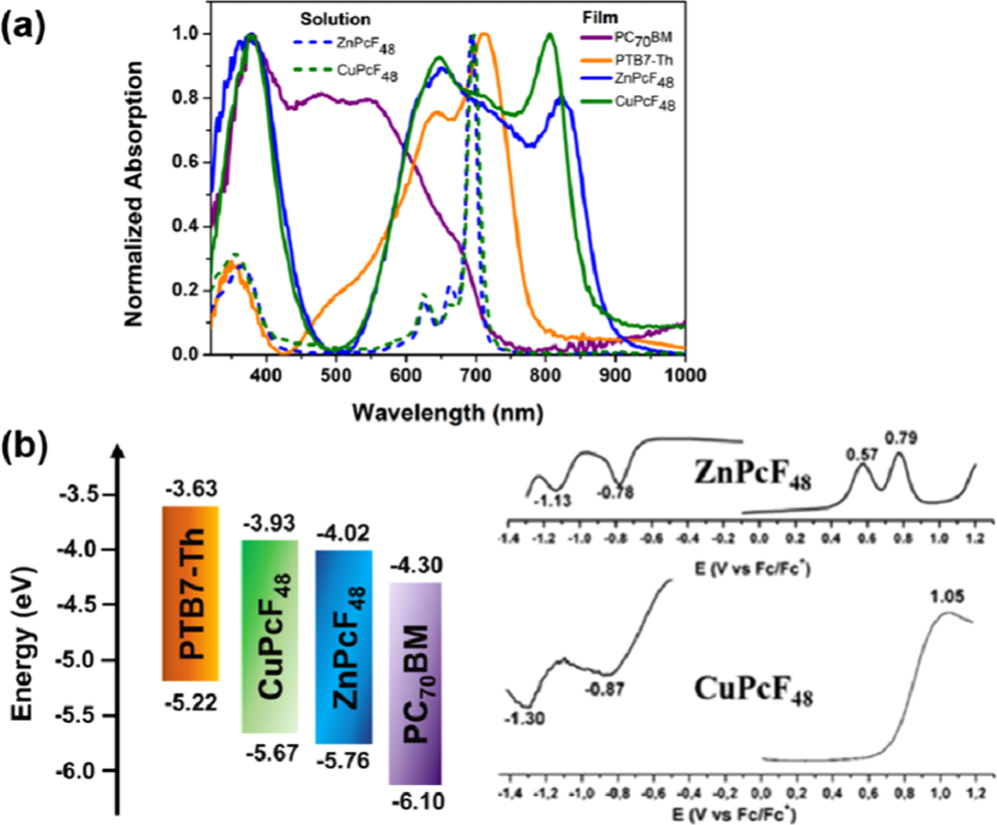
(a) Normalized UV–vis spectra of individual solutions (dashed lines) and thin films (solid lines) and (b) diagram representation of the estimated energy levels for PTB7-Th, CuPcF_48_, ZnPcF_48_, and PC_70_BM (left), calculated from differential pulse voltammetry in DCM (ZnPcF_48_) and THF (CuPcF_48_) using 0.1 M TBAPF_6_ as supporting electrolyte (right).

Just like phthalonitrile **1**, ZnPcF_48_ was not possible to characterize using ^1^H NMR because of the absence of proton signals but it was characterized using ^19^F NMR, ^13^C NMR, and 2D NMR experiments using THF-*d*_8_ as solvent. The ^19^F NMR spectrum shows four different signals, and their assignment was made thanks to different multiplicities and the 2D experiment (see Figures S3 and S4). The ^13^C NMR of ZnPcF_48_ was recorded with and without fluoride decoupling, as shown in Figures S5 and S6, respectively. The carbons most affected by the formation of the phthalocyanine ring are A and H, which appear more deshielded than in the case of phthalonitrile **1** (see Figure S7). The remaining carbons are not as so affected. The existence of fluorine atoms in the nonperipheral positions of the phthalocyanine ring is also demonstrated by signal B in the ^13^C NMR experiment with fluoride coupling (see Figure S6). This signal appears as a doublet of doublets with coupling constants of 4 and 266 Hz, being the latter characteristic of ^13^C–^19^F couplings. In the case of CuPcF_48_, due to the paramagnetic character of copper, this it was not possible to characterize by NMR experiments. Both phthalocyanines were further characterized by FT-IR, HR-MALDI-TOF, and UV–vis experiments. The FT-IR for both phthalocyanines shows the absence of the characteristic peak of C≡N around 2200 cm^–1^, which appears in the FT-IR of phthalonitrile **1** (see Figures S8–S10). The mass experiments show the expected molecular peaks with an isotopic distribution that exactly matched the simulated isotopic patterns (see Figures S11–S13). The UV–vis spectra of the individual thin film and the CHCl_3_ solution of PTB7-Th, ZnPcF_48_, CuPcF_48_, and PC_70_BM are shown in Figure 2a. In solution, ZnPcF_48_ and CuPcF_48_ exhibit the Soret band around 360 nm and the Q band around 694 nm, which are the characteristics of nonaggregated phthalocyanines. Compared with solution absorption spectra, ZnPcF_48_ and CuPcF_48_ films show a broader absorption range and, respectively, perform a red shift to the near-IR absorption spectra of 822 and 806 nm, which suggests the presence of ordered *J*-aggregations in the film.^45^ Besides the near-IR absorption peak, other strong absorption peaks of ZnPcF_48_ and CuPcF_48_ films appear at visible ranges (378 and 652 and 382 and 648 nm), which is complementary to both PTB7-Th and PC_70_BM. The complementary absorption should be beneficial to aid the photon harvesting efficiency, resulting in high short-circuit current density (*J*_SC_), and further improve the efficiency of ternary devices. The complementary absorption spectra of the binary and ternary thin films are displayed in Figures S14 and S15.

Due to the π–π stacking of the molecules in the solution, the oxidation and reduction potentials were not able to measure by cyclic voltammetry. These values were obtained from differential pulse voltammetry (DPV) in DCM and dry THF as solvent, for ZnPcF_48_ and CuPcF_48_, respectively, containing 0.1 M TBAPF_6_ as the supporting electrolyte. As shown in Figure 2b, ZnPcF_48_ has two oxidation potentials at 0.57 and 0.79 V and two reduction potentials at −0.78 and −1.13 V versus Fc/Fc^+^. Meanwhile, CuPcF_48_ shows one oxidation potential at 1.05 V and two reduction potentials at −0.87 and −1.30 V versus Fc/Fc^+^. The estimated LUMO energy levels were calculated according to the equation *E*_LUMO_ = −4.8 – *E*_red_, where *E*_red_ is the first reduction potential. For ZnPcF_48_ and CuPcF_48_, *E*_LUMO_ values are −4.02 and −3.93 eV, respectively. As a consequence of the difficulty to obtain the first oxidation potential of CuPcF_48_, the HOMO energy levels for ZnPcF_48_ and CuPcF_48_ were determined, adding the absorption onset to the *E*_LUMO_ value. These values for ZnPcF_48_ and CuPcF_48_ are −5.76 and −5.67 eV, respectively, as shown in Figure 2b. The energy band gap from the absorption onset was obtained from eq 1
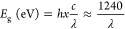
1where λ denotes the absorption edge wavelength expressed in nm, obtained from the offset wavelength derived from the low energy absorption band, as represented in Figure S16.

The HOMO and LUMO energy offsets of PTB7-Th and PC_70_BM are reduced by the addition of ZnPcF_48_ and CuPcF_48_ as third components in a ternary blend, which provides more pathways to enhance the charge transfer of both electrons and holes by forming a cascade energy transfer.^22,46^ By using a photoluminescence instrument (see Figure S17), the emission of MPcF_48_ is moderately overlapped with the absorption of PTB7-Th, suggesting the presence of energy transfer via Forster resonance energy transfer (FRET).^46^ Hence, these results suggest that the subtle change in the metal atom in MPcF_48_ can significantly influence the photophysical and electrochemical properties.

### 2.2 Photovoltaic Properties

**Figure 3:**
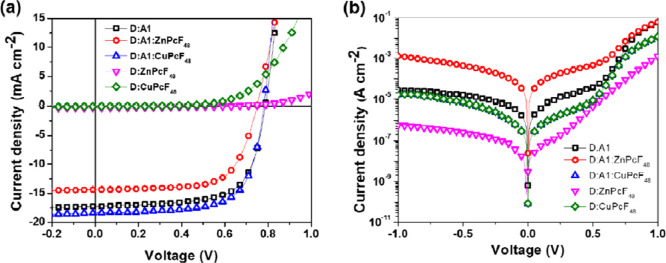
*J*–*V* characteristics of the binary and ternary solar cell devices under (a) AM 1.5G illumination and (b) dark condition.

The device performance of binary and ternary OSCs with different fluorinated phthalocyanine small molecules is thoroughly studied. The OSC devices were fabricated with an inverted configuration of ITO/PFN/active layer/V_2_O_5_/Ag. The ratio of MPcF_48_ in the ternary devices was optimized, as summarized in Tables S1–S3. The ratio of MPcF_48_ to the PTB7-Th:PC_70_BM blend in the active layer was varied from 0 to 12% while keeping the donor-to-acceptor weight ratio constant at 1:1.5. The binary PTB7-Th:MPcF_48_ blend was performed with a donor-to-acceptor weight ratio of 1:1 for further investigation. The current density versus voltage (*J*–*V*) characteristics of the optimized OSC devices based on the PTB7-Th:PC_70_BM (1:1.5), PTB7-Th:PC_70_BM:ZnPcF_48_ (1:1.47:0.03), PTB7-Th:PC_70_BM:CuPcF_48_ (1:1.47:0.03), PTB7-Th:ZnPcF_48_ (1:1), and PTB7-Th:CuPcF_48_ (1:1) bulk heterojunction blends under AM 1.5G illumination (100 mW·cm^–2^) and dark illumination are shown in Figure 3. The device parameters such as *J*_SC_, open-circuit voltage (*V*_OC_), fill factor (FF), and PCE are summarized in Table 1. To make a good data statistic, the extracted data were obtained from over 10 devices. As shown in Figure 3a and Table 1, the PC_70_BM-based binary reference OSCs show a max PCE of 9.23% with a *J*_SC_ of 16.82 mA·cm^–2^, a *V*_OC_ of 0.78 V, and a FF of 68.22%. Both MPcF_48_-based binary OSCs show poor device performance compared to PC_70_BM-based binary devices. CuPcF_48_-based binary OSCs show a max PCE of 0.01% with a *J*_SC_ of 0.06 mA·cm^–2^, a *V*_OC_ of 0.32 V, and an FF of 0.24%. Despite having low performance, ZnPcF_48_-based binary OSCs have 1 order of magnitude higher PCE than those of CuPcF_48_-based binary OSCs with a max PCE of 0.07%, a *J*_SC_ of 0.27 mA·cm^–2^, a *V*_OC_ of 0.33 V, and an FF of 0.50%. This may arise from a larger ΔHOMO of PTB7-Th/ZnPcF_48_ (0.54 eV) than that of PTB7-Th/CuPcF_48_ (0.45 eV). As reported by Yang *et al.*, minimizing the energy level offset between a donor and non-fullerene acceptor may provide sufficient driving force for more efficient exciton dissociation, thus increasing the PCE.^47^ On the other hand, ZnPcF_48_ TOSCs show a max PCE of 7.21% with a *J*_SC_ of 14.25 mA·cm^–2^, a *V*_OC_ of 0.78 V, and an FF of 64.47%. Even though ZnPcF_48_ exhibits complementary absorption and energy cascade transfer for PTB7-Th and PC_70_BM, its lower device performance than those of PC_70_BM binary reference devices may be attributed to the tendency of ZnPcF_48_ to solubilize in either the PTB7-Th or PC_70_BM phase rather than at the PTB7-Th:PC_70_BM interface.^48^ By using contact angle measurement, we found that ZnPcF_48_ materials are likely to be miscible in the acceptor phase (see Table S4). We then explored CuPcF_48_ as the third component in TOSCs. A max PCE of 9.92% with a *J*_SC_ of 17.90 mA·cm^–2^, a *V*_OC_ of 0.78 V, and a FF of 68.54% was achieved. It is demonstrated that their *J*_SC_ and FF are higher than those of reference binary devices. The enhancement in the *J*_SC_ is attributed to the complementary absorption profile of the ternary active layer (Figures S14 and S15). A slightly higher value of FF for CuPcF_48_ TOSCs as compared to PC_70_BM binary OSCs may be related to the fact that CuPcF_48_ improves electron transport.

**Table 1:** Summary of the Device Performance Parameters of Ternary Organic Solar Cells with Different Fluorinated Phthalocyanine Small Molecules^a^

D:A_1_:A_2_	*J*_SC_ (avg)/*J*_calc._^b^ [mA·cm^–2^]	*V*_OC_ (avg) [V]	FF (avg) [%]	PCE (avg/best) [%]
D:A_1_	16.82 (16.00)	0.78	68.22	9.01 (9.23)
D:A_1_:ZnPcF_48_	14.25 (13.88)	0.77	64.47	7.12 (7.21)
D:A_1_:CuPcF_48_	17.90 (17.31)	0.78	68.54	9.57 (9.92)
D:ZnPcF_48_	0.27 (0.25)	0.33	0.50	0.03 (0.07)
D:CuPcF_48_	0.06 (0.06)	0.32	0.24	0.005 (0.006)

a Average values were calculated from over 10 devices.

b *J*_calc._ values in parentheses are the integrated current densities calculated from EQE curves.

Figure 3b depicts the *J*–*V* characteristic measured in the dark, which gives a further understanding of leakage current and series and shunt resistance in the device performance. The extracted device performance parameters are summarized in Table S5. At the reverse and low forward voltages, the shunt resistance (*R*_Sh_) was dominated, which is correlated with the leakage current of the devices. The value of *R*_Sh_ for ZnPcF_48_ TOSCs is the lowest among other binary TOSCs and CuPcF_48_ TOSCs. As a result, ZnPcF_48_ TOSCs have the highest leakage current, which can dominate the photocurrent and thereby reduce the light current. On the other hand, upon adding CuPcF_48_ in the TOSCs, the leakage current greatly decreases at negative voltages compared to those of binary OSCs. At a region of an applied voltage of around 0.5–1.0 V, CuPcF_48_ TOSCs and PC_70_BM binary devices have similar series resistance (*R*_S_) values and lower than that of ZnPcF_48_ TOSCs. Similar *R*_Sh_ values are also observed in CuPcF_48_ TOSCs and PC_70_BM binary devices, resulting in their similar FF values. A lower FF value of ZnPcF_48_ TOSCs is observed with its much lower *R*_Sh_ value. It suggests that the parasitic series and shunt resistance effect may contribute to the change of FF in TOSCs.

**Figure 4:**
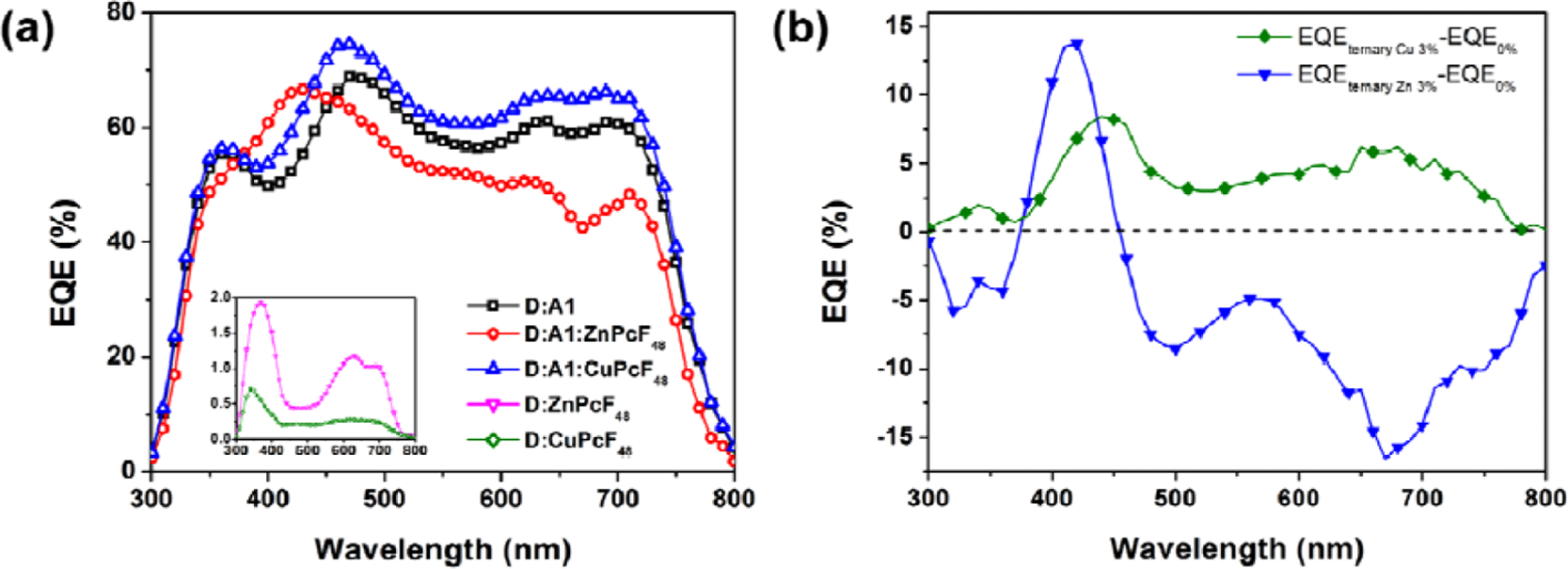
(a) EQE spectra of binary bulk heterojunction OSCs and ternary bulk heterojunction OSCs with two different third components and (b) the ΔEQE values (*y*-axis) of ternary devices with respect to the PC_70_BM binary device.

To gain further insights into enhanced *J*_SC_ values for CuPcF_48_-based ternary systems, the external quantum efficiency (EQE) spectra were measured, as shown in Figure 4a. Binary PTB7-Th:PC_70_BM, PTB7-Th:ZnPcF_48_, and PTB7-Th:CuPcF_48_ bulk heterojunction solar cells show maximum EQE values of 68.93, 1.93, and 0.71% at wavelengths of 470, 370, and 340 nm, respectively. Meanwhile, the maximum EQE value of 66.68% for ZnPcF_48_ TOSCs is lower than that for binary PTB7-Th:PC_70_BM, and the EQE peak is shifted to a lower wavelength at 430 nm. It indicates that the addition of ZnPcF_48_ as the third component in PTB7-Th:PC_70_BM-based OSCs contributes to the deterioration of the device performance, as shown clearly in Figure 4b, by taking ΔEQE (defined as EQE_ternary_ – EQE_binary_). The contribution of ZnPcF_48_ as the third component in the photon-to-electron conversion capability is can only be seen in a wavelength range of 370–450 nm. On the contrary, CuPcF_48_ TOSCs show an enhanced EQE response in the whole wavelength range of 300–800 nm. The highest value of the EQE response of corresponding CuPcF_48_ TOSCs reaches 74.64% at 470 nm. The escalation of the EQE values for CuPcF_48_ TOSCs together with increased *J*_SC_ contributes to the FRET effects, which signify that more excitons are generated due to the enhanced absorbed photons in the ternary photoactive layer. These results suggest that the central metal atom substitution in fluorinated phthalocyanines for the third component in TOSCs apparently affects the photon harvesting efficiency. It is worth mentioning that the integrated current densities *J*_SC_ for binary and ternary devices are in good agreement with the measured *J*_SC_ values from *J*–*V* curves with a maximum error of less than 8%.

**Figure 5:**
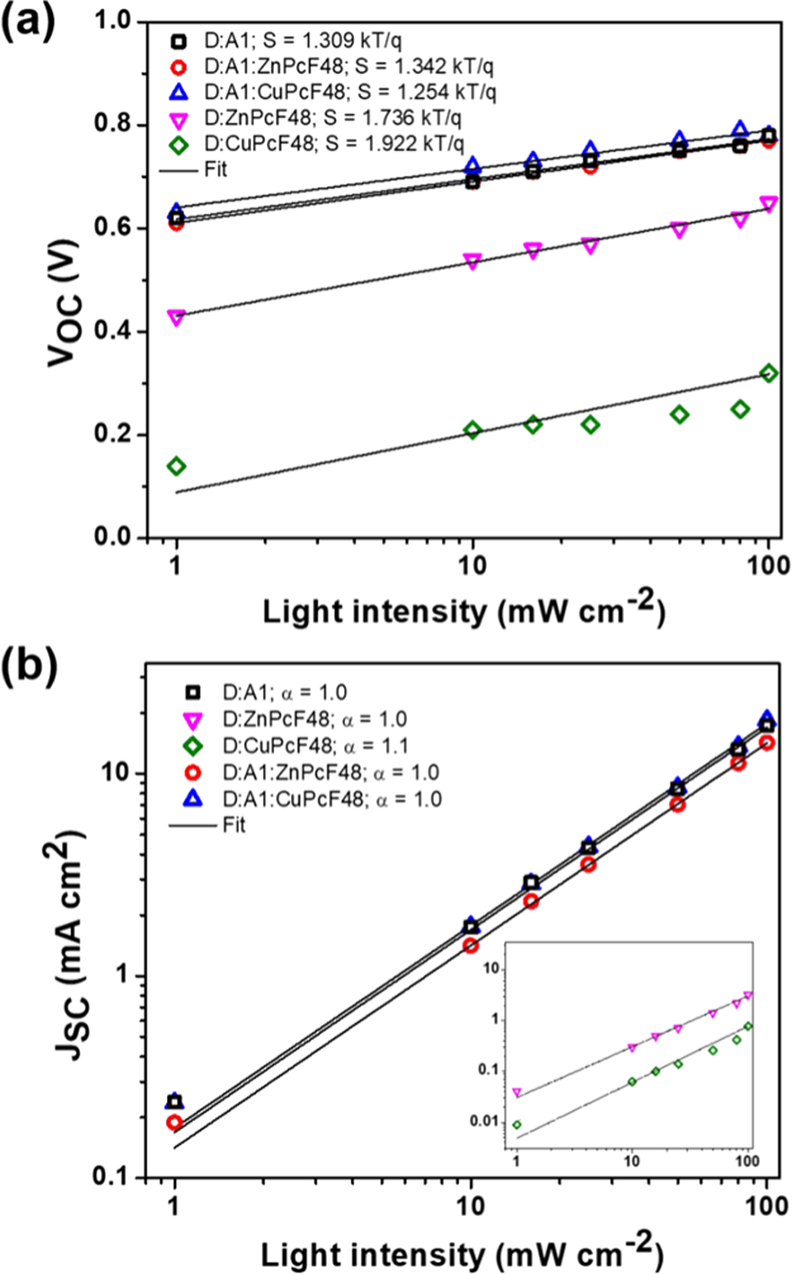
(a) *V*_OC_ and (b) *J*_SC_ versus light intensity of binary and ternary films.

The effects of fluorinated zinc and copper phthalocyanines on the charge recombination dynamics were extracted by analyzing *V*_OC_ and *J*_SC_ as a function of light intensity (*P*_light_). The plot *V*_OC_ versus the natural logarithm of *P*_light_ provides information on the degree of trap-assisted recombination under *V*_OC_ conditions. Typically, a slope (*S*) equal to 1 *kT*/*q* suggests the presence of bimolecular recombination, whereas an *S* ranging between 1 and 2 (1 *kT*/*q* ≤ *S* ≤ 2 *kT*/*q*) indicates the presence of monomolecular recombination and trap-assisted recombination losses.^26,49^Figure 5a shows the plot *V*_OC_ versus ln *P*_light_ for binary devices based on PTB7-Th:PC_70_BM, PTB7-Th:ZnPcF_48_, and PTB7-Th:CuPcF_48_ and ternary OSCs based on PTB7-Th:PC_70_BM:ZnPcF_48_ and PTB7-Th:PC_70_BM:CuPcF_48_. The slope values were calculated to be 1.31 *kT*/*q*, 1.74 *kT*/*q*, 1.92 *kT*/*q*, 1.34 *kT*/*q*, and 1.25 *kT*/*q*, respectively. These results suggest that the trap-assisted recombination losses can be suppressed by adding a small amount of CuPcF_48_ in TOSCs. Consequently, the *J*_SC_ and FF of the ternary OSC PTB7-Th:PC_70_BM:CuPcF_48_ simultaneously increase as compared to those of the binary system made of PTB7-Th:PC_70_BM. However, the higher *S* value of the PTB7-Th:PC_70_BM:ZnPcF_48_ OSC indicates that adding a small concentration of ZnPcF_48_ results in a higher trap-assisted recombination degree, resulting in lower *J*_SC_ and FF as compared to those of the binary system (see Table 1).

Figure 5b shows the power-law function *J*_SC_ ∝ *P*_light_^α^, where α represents the bimolecular recombination intensity. An α value equal to unity (α = 1) indicates that bimolecular recombination does not take place within the devices.^50,51^ The calculated α values are 1 for all devices, excluding the device PTB7-Th:CuPcF_48_ with α = 1.1. The results indicate that there is no bimolecular recombination in all devices.

**Figure 6:**
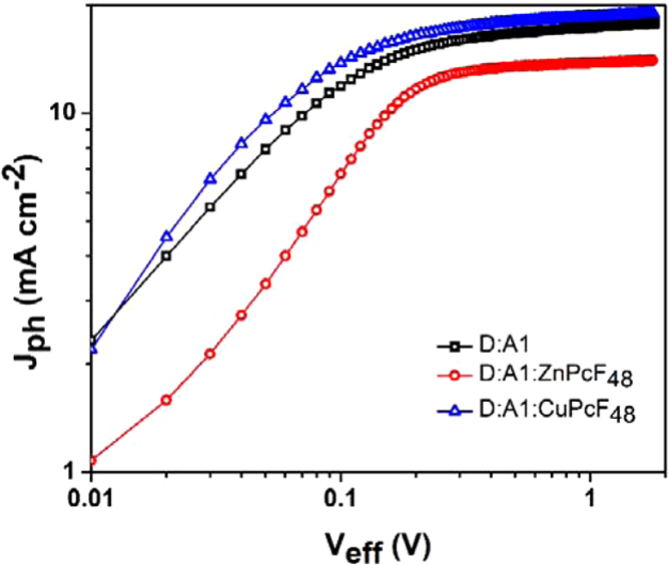
Variation of photocurrent (*J*_Ph_) with effective voltage (*V*_eff_) in PC_70_BM binary and ternary solar cell devices.

To gain insights into the influence of zinc and copper phthalocyanines on the charge dynamics, we measured the photocurrent (*J*_Ph_) as a function of effective voltage (*V*_eff_). *J*_Ph_ is defined as the difference between the current density measured under 1 sun illumination and that measured under dark conditions (*J*_Ph_ = *J*_L_ – *J*_D_). On the other hand, *V*_eff_ is defined as *V*_eff_ = *V*_0_ – *V*_app_, where *V*_0_ is the voltage at *J*_Ph_ = 0 and *V*_app_ is the applied voltage. Figure 6 shows the *J*_Ph_ versus *V*_eff_ plot for the binary OSC based on PTB7-Th:PC_70_BM and the ternary OSCs PTB7-Th:PC_70_BM:ZnPcF_48_ and PTB7-Th:PC_70_BM:CuPcF_48_. For PTB7-Th:ZnPcF_48_- and PTB7-Th:CuPcF_48_-based devices, the saturation current could not be reached even at bias >1 V, suggesting severe charge recombination phenomena (see Figures S18–S21). The saturation current density (*J*_sat_) is extracted from Figure 3a, where *J*_Ph_ saturates at a high *V*_eff_ of over 1.5 V for binary and ternary devices. The maximum charge generation rate (*G*_max_) is defined as *J*_sat_ = *qLG*_max_, where *q* is the elementary charge and *L* is the thickness of the binary or ternary organic layer. Besides, the exciton dissociation efficiency (η_diss_) is calculated by the *J*_Ph_/*J*_sat_ value under the short-circuit condition, whereas the charge carrier collection efficiency (η_coll_) is defined by *J*_Ph_/*J*_sat_ under the maximum power point condition. Table S6 summarizes the values of *G*_max_, η_diss_, and η_coll_ for the binary OSC PTB7-Th:PC_70_BM and the ternary OSCs PTB7-Th:PC_70_BM:ZnPcF_48_ and PTB7-Th:PC_70_BM:CuPcF_48_. In the ternary OSC PTB7-Th:PC_70_BM:CuPcF_48_, *G*_max_ is slightly higher than those of the binary devices, while the *G*_max_ of PTB7-Th:PC_70_BM:ZnPcF_48_ is lower than that of the binary system; however, *G*_max_ for binary–ternary OSCs are almost in the same order of magnitude. Moreover, the PTB7-Th:PC_70_BM:CuPcF_48_ OSC exhibited the highest values of η_diss_ and η_coll_, indicating that adding 3% CuPcF_48_ into a PTB7-Th:PC_70_BM system allows improving the charge dynamics of the ternary system. These results agree with the improved *J*_SC_ and FF of the PTB7-Th:PC_70_BM:CuPcF_48_ OSC in comparison to those of PTB7-Th:PC_70_BM.

### 2.3 Thin-Film Surface Properties

**Figure 7:**
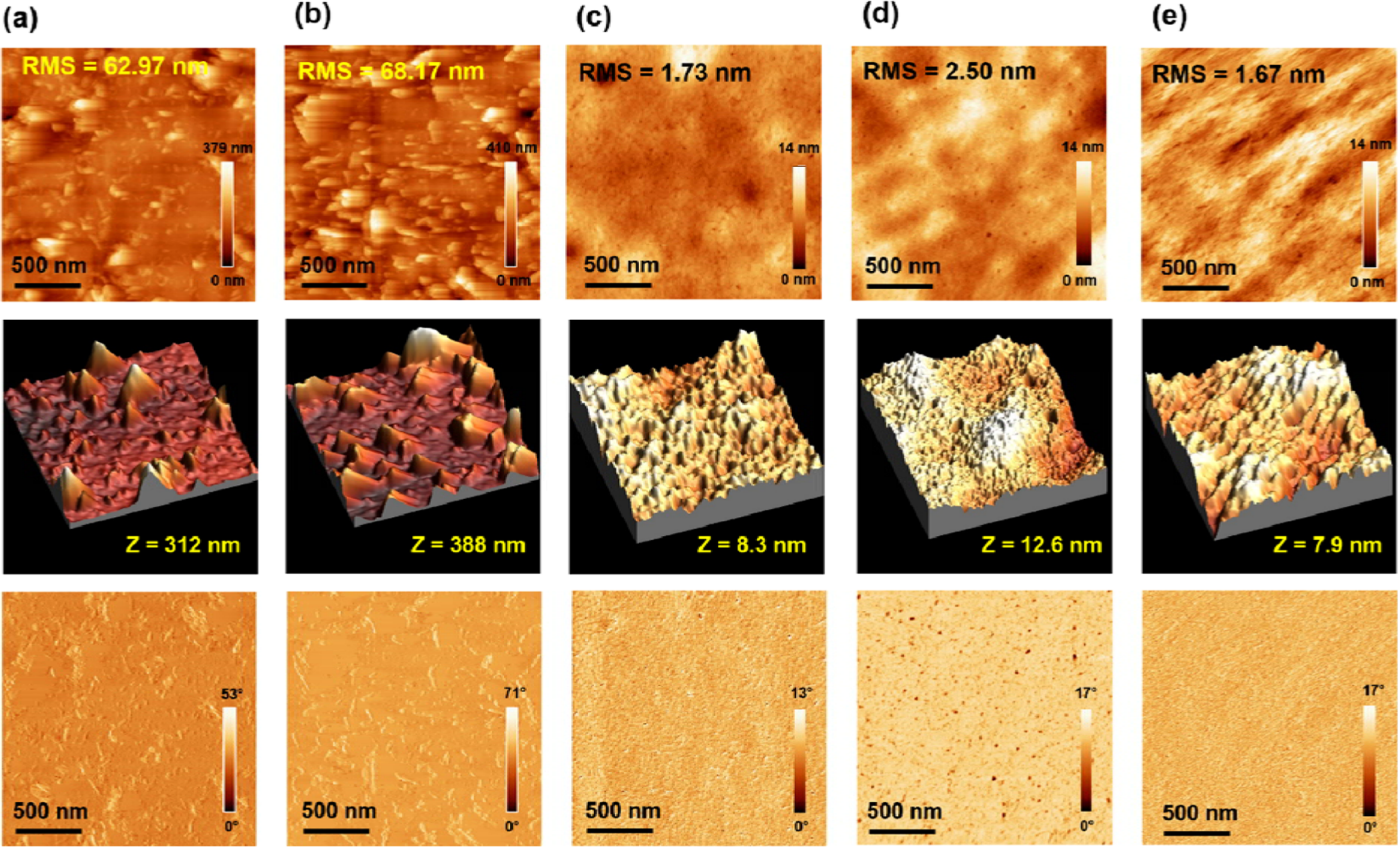
AFM height images (top), AFM three-dimensional height images (middle), and AFM phase images (bottom) (2 × 2 μm^2^) of binary (a) D:ZnPcF_48_, (b) D:CuPcF_48_, and (c) D:A_1_ and ternary (d) D:A_1_:ZnPcF_48_ and (e) D:A_1_:CuPcF_48_.

Even though ZnPcF_48_ and CuPcF_48_ have the same value of the energy band gap (*E*_g_ = *E*_HOMO_ – *E*_LUMO_) of 1.74 eV and similar HOMO and LUMO values, the ZnPcF_48_-based binary OSC showed an order of magnitude higher efficiency when paired with PTB7-Th than those of CuPcF_48_. The reason behind this may correlate with the different morphology of the bulk film, as shown in Figure 7. The tapping mode atomic force microscopy (AFM) measurements were performed to investigate the bulk surface morphology changes by introducing MPcF_48_ to the PTB7-Th:PC_70_BM-blend system. The corresponding AFM height and phase images for the PTB7-Th:MPcF_48_ blend are shown in Figure 7a,b (the top part is two-dimensional images, the middle part is three-dimensional images, and the bottom part is the phase images). Both MPcF_48_-based binary blends show a coarse, distinctive phase-separated morphology with a large island-like domain. Larger domain sizes on the photoactive layer of OSCs are not favorable for charge transport or exciton diffusion, thus explaining why MPcF_48_ binary OSCs have inferior device performance (see Table 1). Even though both MPcF_48_ binary blends have a large domain and very rough surfaces, the ZnPcF_48_-based binary blend shows clearly less aggregation because ZnPcs are known to disperse better in solution than CuPcs.^52^ Compared to the PTB7-Th:CuPcF_48_ blend, the PTB7-Th:ZnPcF_48_ blend shows a lower root-mean-square (RMS) roughness of ∼63 nm, less aggregation with smaller hills, and a smaller height difference, as shown in its corresponding 3D height and phase images. This result is in good agreement with the higher performance of the ZnPcF_48_-based binary OSC than the CuPcF_48_-based binary OSC. Due to the poor morphology of the MPcF_48_ binary film, they are likely to play a bigger role than small energetic differences.

Figure 7c displays the morphology of the binary reference PTB7-Th:PC_70_BM blend film featuring a smooth crystallized-like domain with an RMS roughness of 1.72 nm. In the case of a ternary system, the addition of 3% ZnPcF_48_ as the third component in the PTB7-Th:PC_70_BM blend results in an increased RMS roughness of 2.5 nm and a larger height difference of 12.6 nm, as shown in Figure 7d. Moreover, the formation of the homogeneous dispersity of pinholes on the ZnPcF_48_ ternary blend film is clearly observed in the phase image, which is not beneficial to facilitate charge transport. On the other hand, the incorporation of 3% CuPcF_48_ in the PTB7-Th:PC_70_BM blend effectively decreases RMS to 1.67 nm and results in a finer interpenetrating bulk heterojunction morphology relative to the PC_70_BM-binary blend, as shown in Figure 7e. The smoother morphology of the PTB7-Th:PC_70_BM:CuPcF_48_ ternary blend compared to the PC_70_BM binary blend may explain the reduced recombination losses and better charge dynamics of CuPcF_48_-based ternary blend compared to the binary blend, which has been reflected in the higher values of FF and *J*_SC_ in the incorporation of CuPcF_48_ as the third component in TOSC devices.

### 2.4 Stability Study

In addition to high device performance, the information about ambient stability is necessarily important for the future commercialization of OSCs. Generally, the ambient photostability of OSCs depends on several factors, such as exposure to humidity and oxygen, intrinsic properties of the organic materials, fullerene dimerization in FA-based OSCs, microstructural stability of NFA-based OSCs, etc. Recent studies about the photostability investigation of ternary OSCs showed that the incorporation of the third component might enhance the photostability of the ternary system by minimizing the molecular structural changes, preserving the crystallinity, deactivating the light-induced trap formation, and balancing the charge carrier mobilities in their active layers upon photodegradation exposure.^53,54^ We believe that the incorporation of MPcF_48_ as the third component in TOSCs may affect the device photostability. Thus, we performed the photostability study of nonencapsulated binary and ternary devices exposed under continuous AM 1.5G illumination at the open-circuit condition in an ambient atmosphere (room temperature and relative humidity = 50–60%).

**Figure 8:**
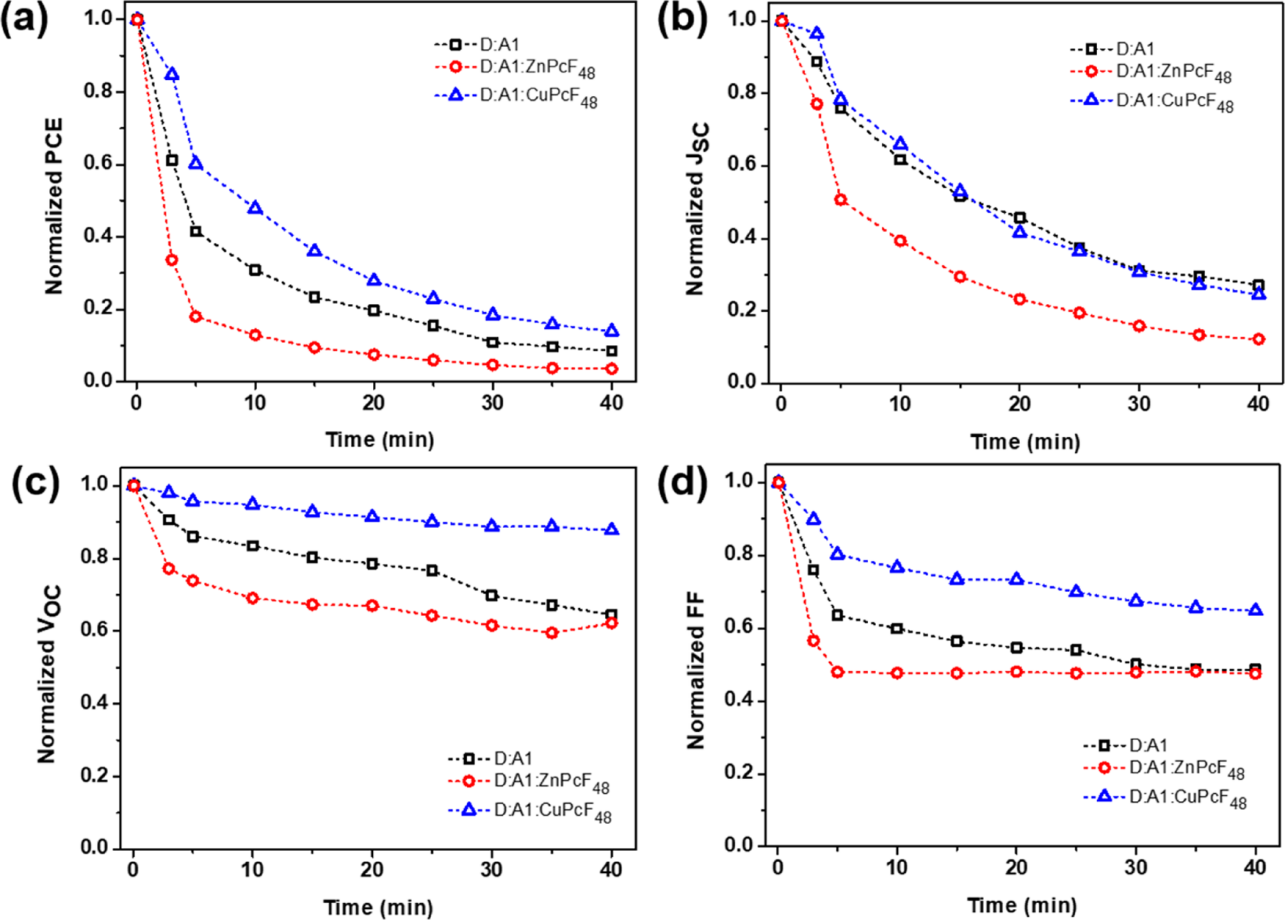
Normalized (a) PCE, (b) *J*_SC_, (c) *V*_OC_, and (d) FF of PC_70_BM binary, ZnPcF_48_ ternary, and CuPcF_48_ ternary devices over photoaging time.

The evolution results of photovoltaic parameters for all devices under the ambient photodegradation test are presented in Figure 8. ZnPcF_48_ TOSCs suffer the most in degradation with more than 95% efficiency loss after 40 min of illumination, as shown in Figure 8a. On the other hand, the PCE of PC_70_BM binary OSCs is reduced by 90% from its original value. The most stable device is CuPcF_48_ TOSCs, with a PCE decay of almost 85%. Reasonable photoaging decay was noticed for all photovoltaic parameters in all devices, as shown in Figure 8b–d. It is obvious to see that *J*_SC_ decay in all photodegraded devices has similar behavior with PCE decay behavior, suggesting that *J*_SC_ is the main contributor to the observed PCE loss. Moreover, it is observed that FF decay also contributes to the PCE loss after *J*_SC_ decay. A relatively higher *J*_SC_ and FF loss in photodegraded ZnPcF_48_ TOSCs may be attributed to the morphological instabilities of bulk heterojunction.^55^ On the other hand, the addition of 3% CuPcF_48_ in the binary PTB7-Th:PC_70_BM blend can moderately enhance the ambient photostability, verified by the relatively lowest *V*_OC_ and FF loss. This result indicates that the metal atom substitution strategy in the third component of TOSCs affects the photostability of OSCs. Further study needs to be carefully carried out to go more in depth into the main degradation mechanism caused by the metal atom substitution of MPcF_48_ as the third component in TOSCs for a full application of the ternary strategy in the future.

## 3 Conclusions

In summary, we have successfully synthesized fluorinated zinc and copper phthalocyanines (ZnPcF_48_ and CuPcF_48_) as new third components in ternary OSCs with a device architecture of ITO/PFN/PTB7-Th:PC_70_BM:MPcF_48_/V_2_O_5_/Ag. The introduction of MPcF_48_ with different central atoms has an influence on the electronic structure, optical absorption, energy level alignment, charge transport and recombination, and blend film morphology of TOSCs. Among MPcF_48_ derivatives, CuPcF_48_ has the highest PCE of up to 9.92%, due to its better morphology, improved exciton dissociation, better charge carrier collection efficiency, and more suppressed trap-assisted recombination, thus making it a promising new NFA material for OSC applications. Further studies are in progress to optimize the devices using new CuPcs as the third component of TOSCs to increase PV performance and stability.

## 4 Experimental Method

### 4.1 Materials

An indium tin oxide (ITO)-patterned glass substrate with a resistivity of 10 Ω·sq^–1^ was purchased from PsiOTec Ltd. Poly[(9,9-bis(3′-(*N*,*N*-dimethylamino)propyl)-2,7-fluorene)-*alt*-2,7-(9,9-dioctylfluorene)] (PFN) and vanadium oxide (V_2_O_5_) as electron and hole transport layer materials were obtained from 1-Material Inc. and Sigma-Aldrich, respectively. Active layer materials used were based on the polymer donor poly[4,8-bis(5-(2-ethylhexyl)thiophen-2-yl)benzo[1,2-b;4,5-b′]dithiophene-2,6-diyl-*alt*-(4-(2-ethylhexyl)-3-fluorothieno[3,4-*b*]-thiophene)-2-carboxylate-2,6-diyl] (PTB7-Th) and the fullerene acceptor [6,6]-phenyl-C_70_-butyric acid methyl ester (PC_70_BM), which were purchased from 1-Material Inc. The third components of the non-fullerene acceptors of zinc- and copper-fluorinated phthalocyanines were synthesized. The chemical structure of active materials in the ternary system is shown in Figure 1. High-purity silver (Ag) was purchased from Testbourne Ltd. All solvents were used as received unless otherwise noted.

### 4.2 Synthesis of 3,6-Difluoro-4,5-bis(pentafluorophenoxy)phthalonitrile **1**

Tetrafluorophthalonitrile (2.0 g, 10 mmol), pentafluorophenol (3.7 g, 20 mmol), and KF (4.0 g, 69 mmol) were dissolved in 20 mL of acetone under an inert atmosphere and stirring at room temperature for 3 days. The solution was filtered, dried under MgSO_4_, filtered again, and concentrated under vacuum. The white solid was washed with methanol yielding 3.0 g (57%) of 3,6-difluoro-4,5-bis(pentafluorophenoxy)phthalonitrile **1**. ^13^C NMR (75 MHz, CDCl_3_): δ = 152.0 (dd, *J* = 265, 3.9 Hz), 142.5–138.9 (m), 141.6–141.2 (m), 140.0–136.0, 138.0–137.0 (m), 130.3–130.0 (m), 108.6, 102.3–101.9 (m). MS-MALDI-TOF *m*/*z*: 527.872. ν_max_(KBr)/cm^–1^: 2247 (C≡N), 1520, 1478, 1397, 1315, 1287, 1164, 1119, 1089, 999. mp = 120 °C.

### 4.3 Synthesis of ZnPcF_48_

Phthalonitrile **1** (300 mg, 0.66 mmol) and Zn(OAc)_2_ (208 mg, 1.14 mmol) were added in a pressure tube and heated at 160 °C overnight. The blue mixture was cooled to room temperature and purified by column chromatography (DCM), yielding 35 mg (11%) of ZnPcF_48_. ^19^F NMR (300 MHz, THF-*d*_8_): δ = −134.8 (s), −157.9 (d, *J* = 23 Hz), −162.5 (t, *J* = 23 Hz), −164.50 (t, *J* = 22 Hz). ^13^C NMR (125 MHz, THF-*d*_8_): δ = 150.5 (s), 148.1–146.0 (dd, *J* = 266, 4 Hz), 143.1–141.1 (dd, *J* = 252, 11 Hz), 140.9–138.7 (m), 140.2–139.9 (m), 139.9–138.0 (m), 132.8 (m), 122.6 (m). ^13^C {^19^F} NMR (125 MHz, THF-*d*_8_): δ = 150.5, 147.1, 142.1, 139.9, 139.8, 139.1, 132.8, 122.6. UV–vis (CHCl_3_) λ_max_/nm (log ε): 365 (4.75), 625 (4.54), 664 (4.62), 694 (5.32). HR-MALDI-TOF (dithranol) *m*/*z* for C_80_F_48_N_8_O_8_Zn: calcd, 2175.8359; found, 2175.8419. *ν*_max_(KBr)/cm^–1^: 1520, 1476, 1417, 1322, 1167, 1122, 1002.

### 4.4 Synthesis of CuPcF_48_

Phthalonitrile **1** (300 mg, 0.66 mmol) and CuCl_2_ (76 mg, 0.66 mmol) were added in a pressure tube and heated at 160 °C overnight. The blue mixture was cooled to room temperature, dissolved in DCM, filtered, and concentrated under vacuum. The green solid was washed with MeOH and purified by Soxhlet extraction using THF obtaining CuPcF_48_ (105 mg, 34%). UV–vis (CHCl_3_) λ_max_/nm (log ε): 355 (4.73), 625 (4.50), 664 (4.42), 697 (5.20). HR-MALDI-TOF (dithranol) *m*/*z* for C_80_CuF_48_N_8_O_8_: calcd, 2174.8441; found, 2174.8420. ν_max_(KBr)/cm^–1^: 1520, 1478, 1420, 1321, 1257, 1268, 1125, 1001.

### 4.5 Device Fabrication

The reported OSC devices were fabricated using an inverted structure of ITO/PFN/PTB7-Th:PC_70_BM:MPcF_48_/V_2_O_5_/Ag. The ITO-coated glass substrate was cleaned with a surface-active cleaning agent and diluted in deionized water. The substrate was sonicated in acetone, methanol, and isopropanol anhydrous solvents for 10 min subsequently. The cleaned substrate was dried at 120 °C in an oven for 20 min and transferred to a UV/ozone cleaner for 15 min. PFN as an electron transport layer was dissolved in methanol with the presence of a small amount of acetic acid (2 μL·mL^–1^). The PFN solution with a concentration of 2 mg·mL^–1^ was spin-coated on the cleaned substrate to obtain a 10 nm thickness. The active layer blend solution was prepared by dissolving the donor and acceptor materials in chlorobenzene and 1,8-diiodooctane (97:3% v/v) with a final concentration of 25 mg·mL^–1^. The weight ratio of donor/fullerene acceptor/metallophthalocyanines was 1:1.5-*x*:*x*. The blend solution was stirred and heated at 40 °C overnight, followed by solution aging for 48 h. After the aging time, the blend solution was spin-coated on top of the interlayer at 750 rpm for 30 s to obtain a 100 nm thickness of the active layer. Afterward, the anode layer consisting of 3 nm of V_2_O_5_ and 100 nm of Ag was deposited by thermal evaporation under high vacuum conditions (≤1 × 10^–6^ mbar) with an evaporation rate of 0.01 nm·s^–1^. The effective area for all devices was 0.09 cm^2^. The device optimization results are shown in Tables S1–S3.

### 4.6 Characterization

UV–vis spectra were recorded using a Helios Gamma spectrophotometer. ^13^C NMR spectra were recorded at 25 °C using Bruker AC300 and Bruker AC500 spectrometers. For ^19^F spectra, trifluorotoluene served as an external standard (δ = −63.9 ppm). High-resolution mass spectra were obtained from a Bruker Microflex LRF20 matrix-assisted laser desorption/ionization time-of-flight (MALDI-TOF) using dithranol as a matrix. Differential pulse voltammetry was performed in a conventional three-electrode cell using a μ-AUTOLAB type III potentiostat/galvanostat at a scan rate of 100 mV·s^–1^. A platinum working electrode, a Ag/AgNO_3_ reference electrode, and a platinum wire counter electrode were employed. Ferrocene/ferrocenium was used as an internal standard for all measurements. Photoluminescence measurements were performed on a fluorescence spectrophotometer from Photon Technology International Inc. (Birmingham, NJ) with a Xe lamp used as the excitation light source at room temperature and an excitation wavelength of 610 nm. The electrical properties of OSC devices (*J*–*V* characteristics) were determined using a solar simulator (Abet Technologies model 11000 class type A, Xenon arc) and a Keithley 2400 Source Measure Unit under illumination and dark conditions in the forward voltage sweep direction from −1 to 1 V. The light intensity was calibrated by an NREL certified monocrystalline silicon photodiode. The external quantum efficiency (EQE) measurements were carried out in the forward wavelength sweep direction from 300 to 800 nm using a Lasing IPCE-DC model with a series number of LS1109-232. The film morphologies were studied by AFM using silicon probes with a spring constant of 5 N·m^–1^ in a tapping mode with a resonant frequency of 150 kHz and a tip radius of 1 nm.

## Supporting Information Available

NMR spectra; FT-IR spectra; MS-MALDI-TOF; HR-MALDI-TOF; UV–vis spectra of thin-film blends; photoluminescence spectrum; electrochemical characterization; device optimization; and characterization (PDF)

## Dedication

Dedicated to Prof. Tomás Torres on the occasion of his 70th birthday
