# Supplementary material for: Fluorinated Zinc and Copper Phthalocyanines as Efficient Third Components in Ternary Bulk Heterojunction Solar Cells
Source: ACS Appl Energy Mater. 2021 May 12;4(5):5201–11. doi: 10.1021/acsaem.1c00734 (PMC9677599; doi:10.1021/acsaem.1c00734)
Supplement: Supplementary file 1 — ae1c00734_si_001.pdf [file ae1c00734_si_001.pdf]

Supporting Information

Fluorinated Zinc and Copper Phthalocyanines as Efficient Third Component in Ternary Bulk Heterojunction Solar Cells

Alfonsina Abat Amelenan Torimtubun [a], Jorge Follana-Berná [b], José G. Sánchez [a], Josep Pallarès[a], Ángela Sastre-Santos*[b], and Lluis F. Marsal*[a]*

A. A. A. Torimtubun, Dr. J. G. Sánchez, Prof. J. P. Marzal, Prof. L. F. Marsal
Department of Electric, Electronic and Automatic Engineering
Universitat Rovira i Virgili
Av. Països Catalans 26, 43007 Tarragona, Spain
E-mail: josep.pallares@urv.cat, lluis.marsal@urv.cat

Dr. Jorge Follana-Berná, Prof. Ángela Sastre-Santos
Área de Química Orgánica, Instituto de Bioingeniería
Universidad Miguel Hernández de Elche
Av. de la Universidad s/n. 03202, Elche, Spain
E-mail: asastre@umh.es

Keywords: fluorinated phthalocyanines, third component, ternary organic solar cells, bulk heterojunction, non-fullerene acceptors

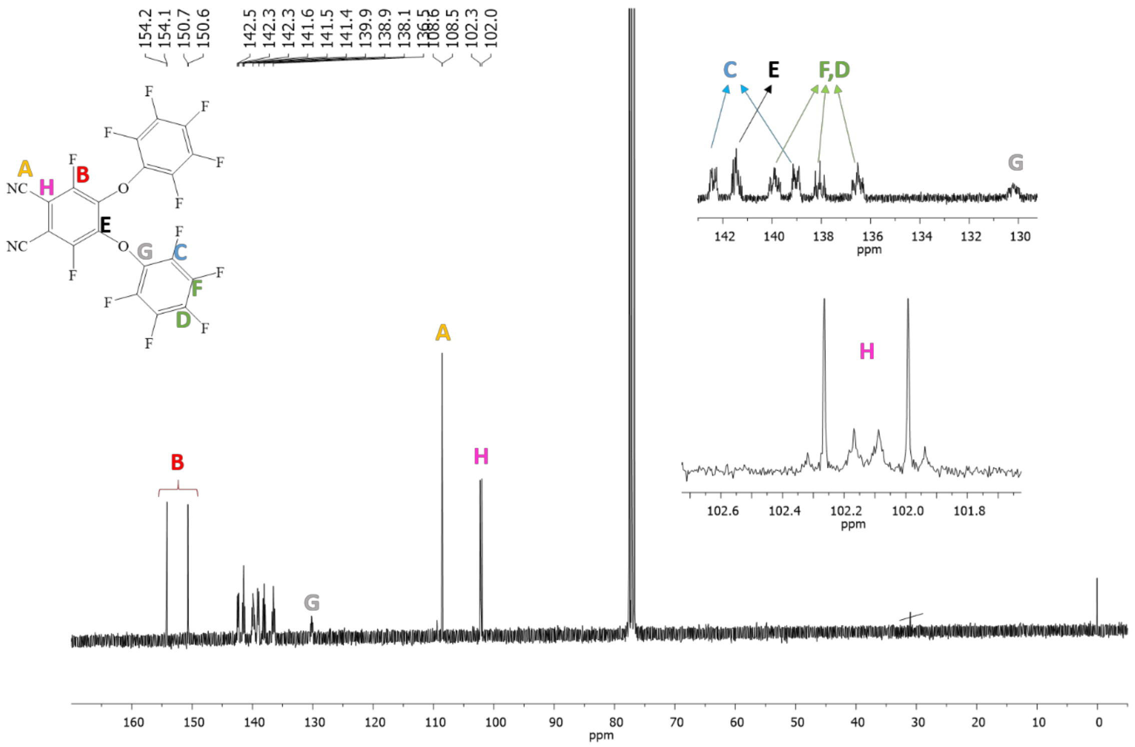

Figure S1. ^{13}C NMR spectrum of phthalonitrile **1** in $CDCl_3$.

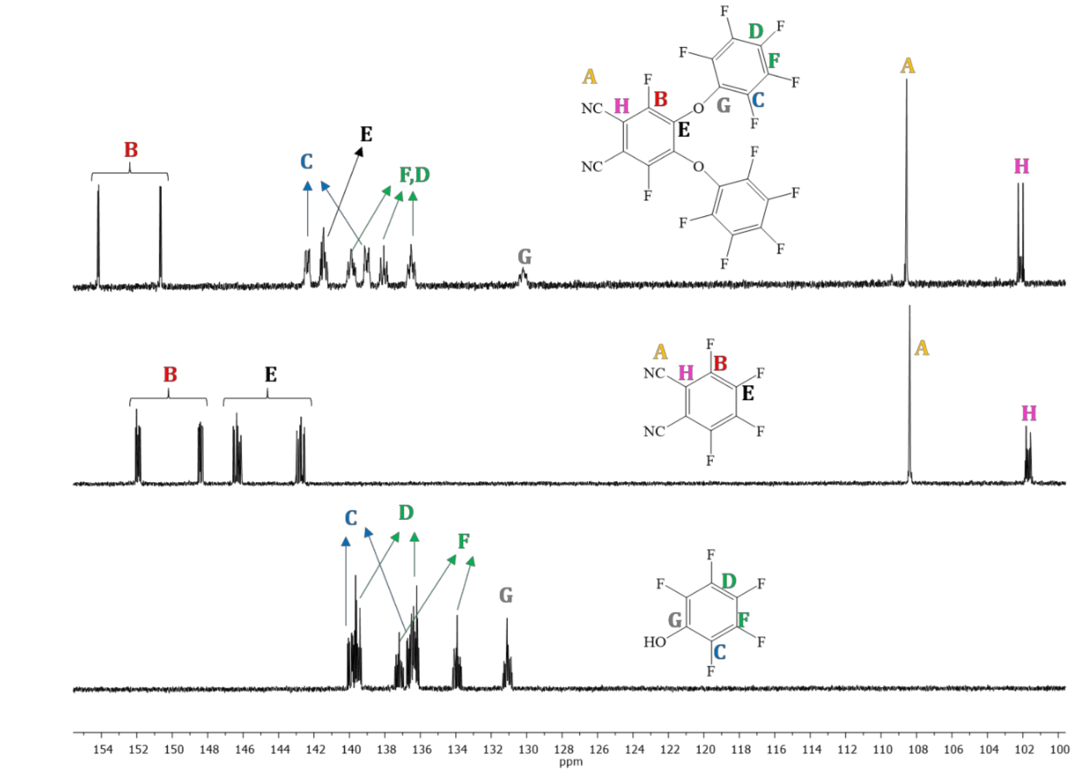

Figure S2. ^{13}C NMR comparison between phthalonitrile **1**, tetrafluorophthalonitrile and pentafluorophenol in $CDCl_3$.

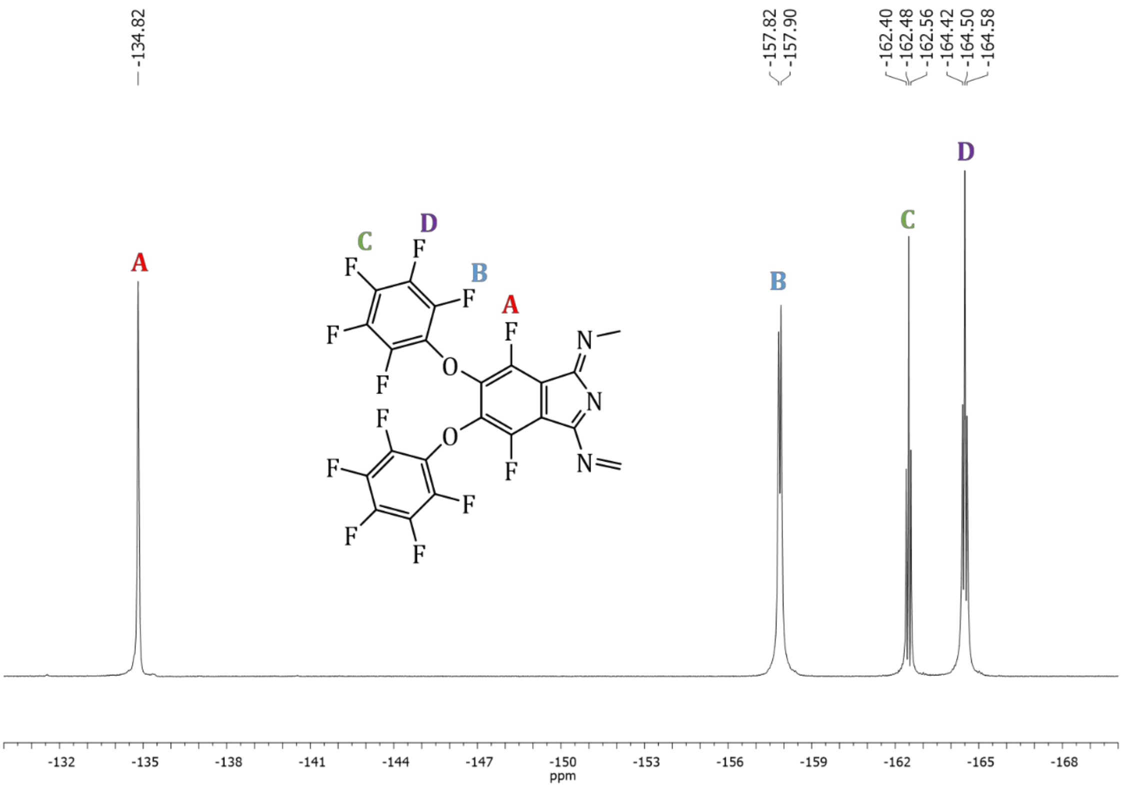

Figure S3. ^{19}F NMR spectrum of **ZnPcF48** in THF-d_8.

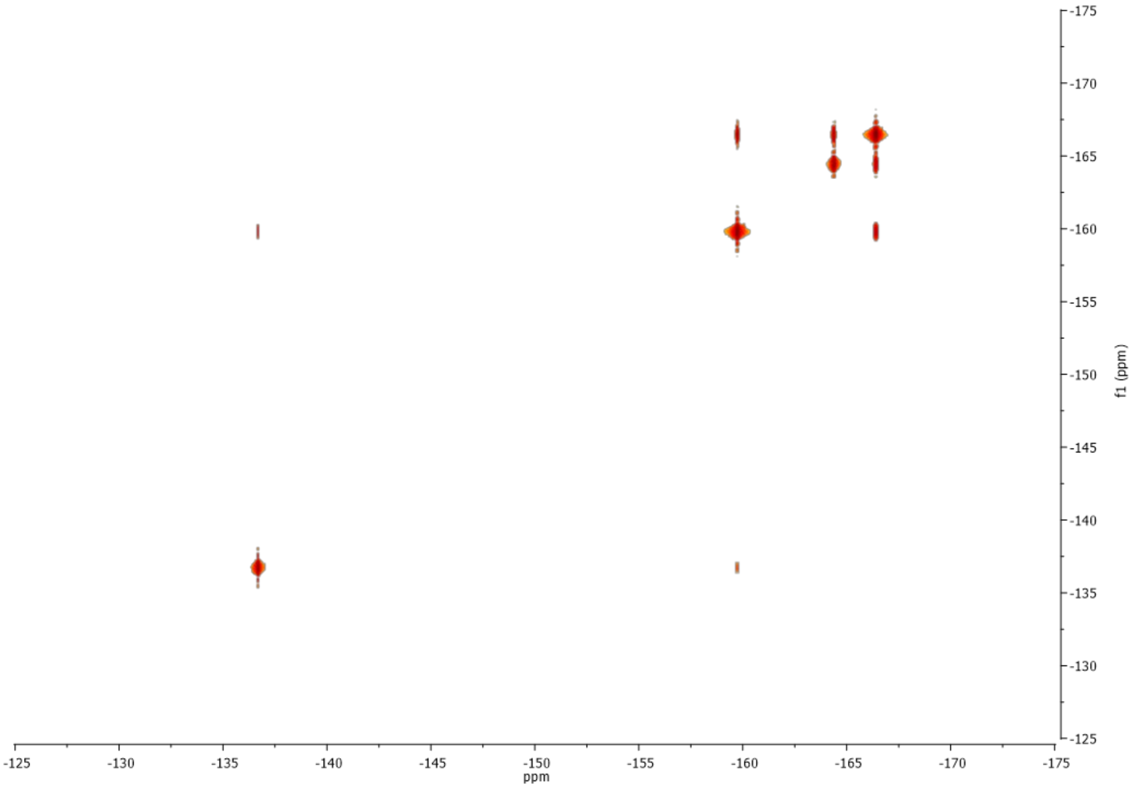

Figure S4. ^{19}F-^{19}F 2D NMR spectrum of **ZnPcF48** in THF-d_8.

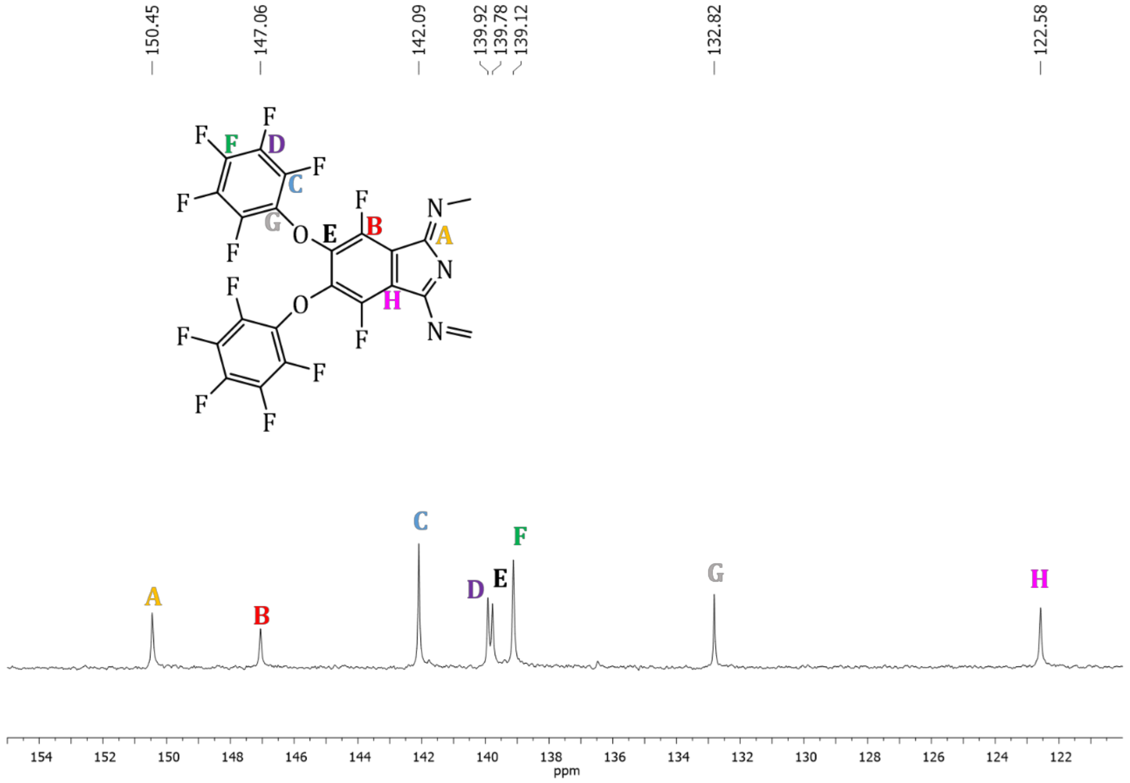

Figure S5. $^{13}C\{^{19}F\}$NMR spectrum of **ZnPcF48** in THF-d_8.

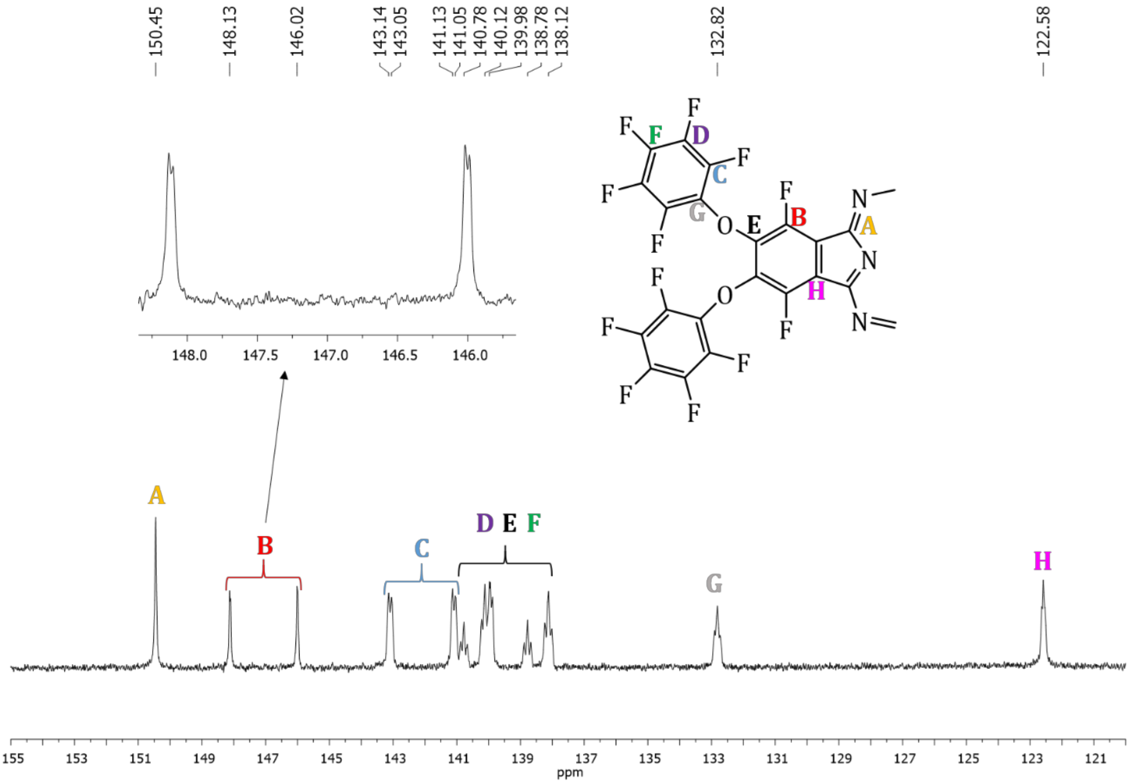

Figure S6. ^{13}C NMR spectrum of **ZnPcF48** in THF-d_8.

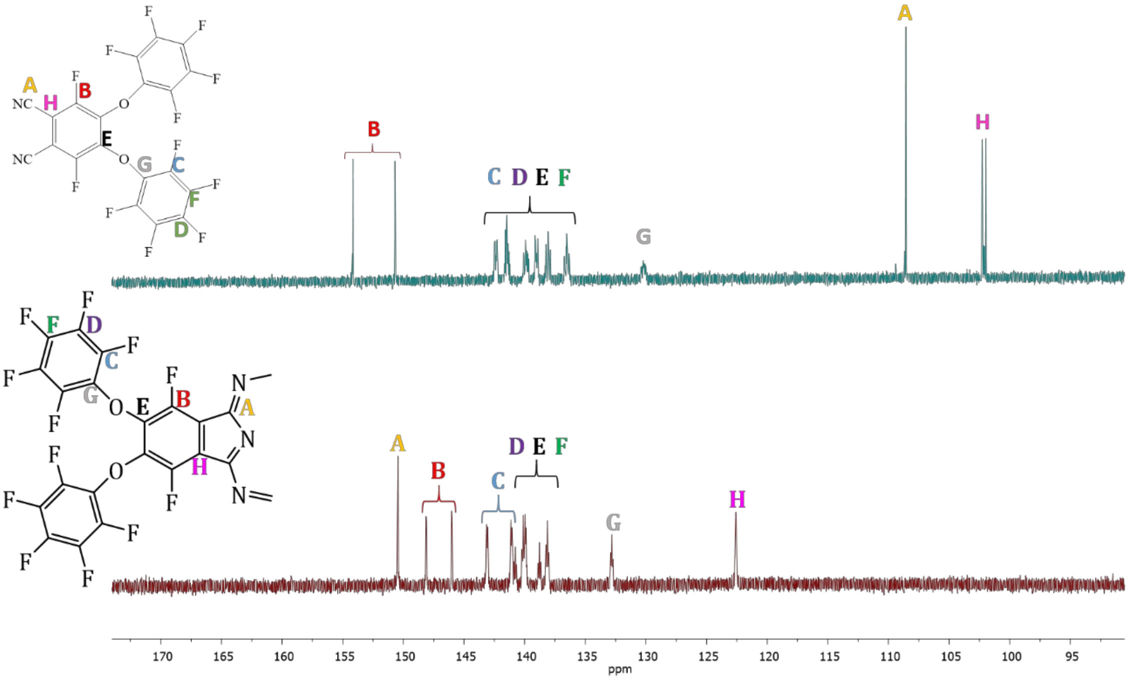

Figure S7. ^{13}C NMR comparison between phthalonitrile **1** and **ZnPcF48**.

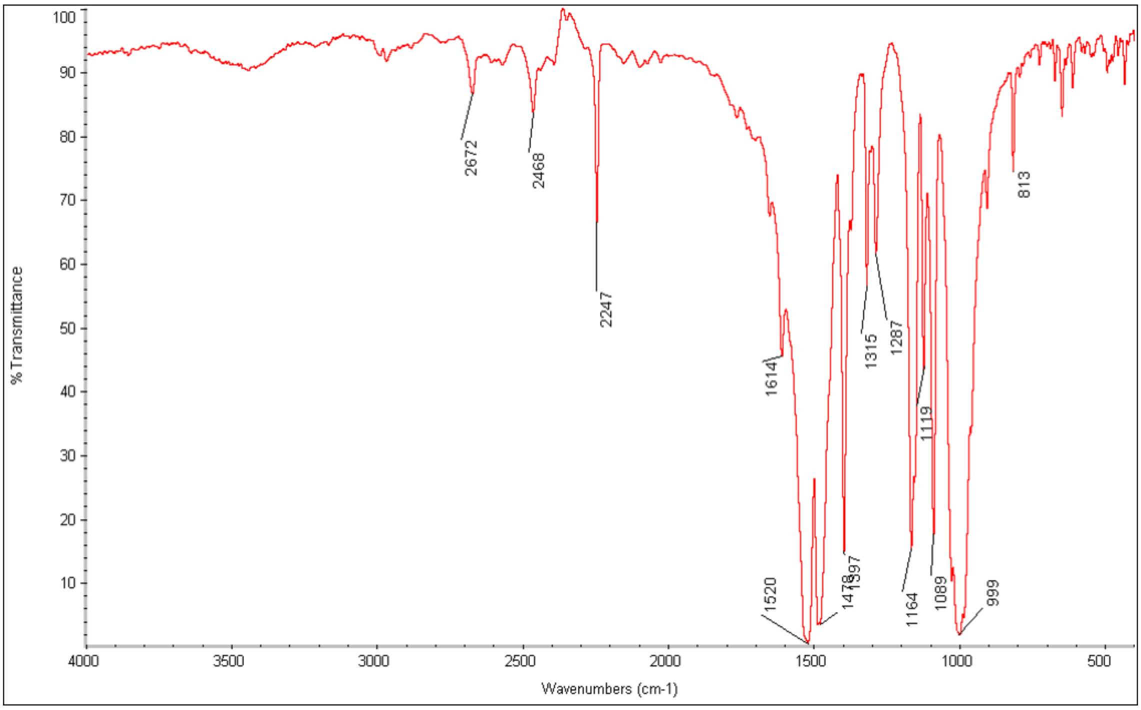

Figure S8. FT-IR spectrum of phthalonitrile **1**.

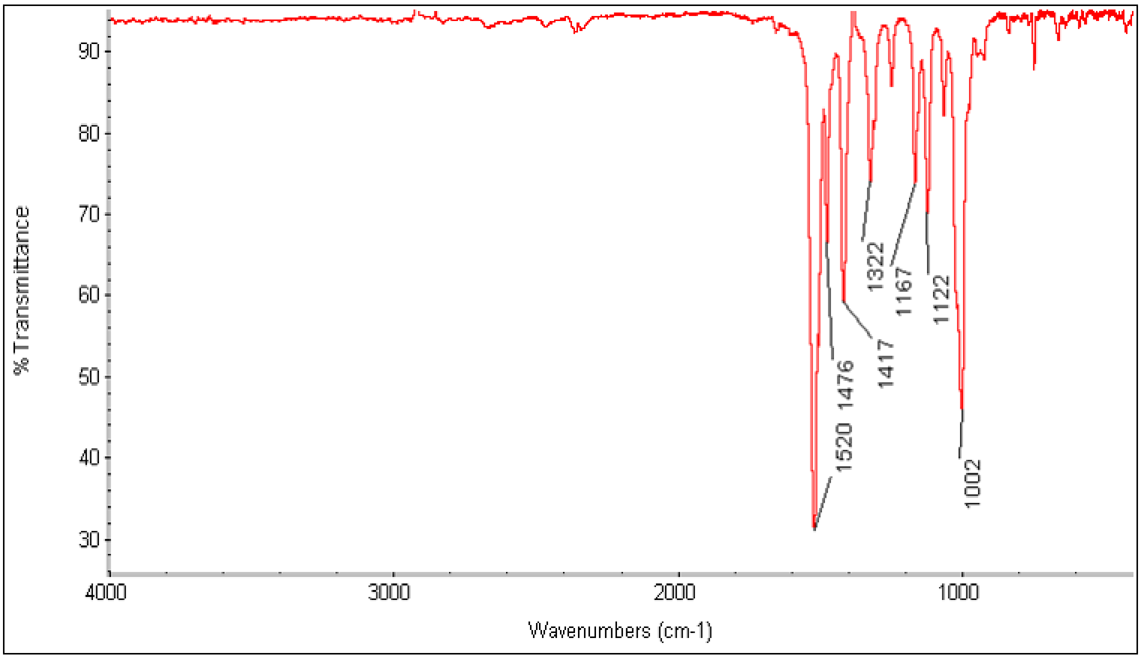

Figure S9. FT-IR spectrum of **ZnPcF48**.

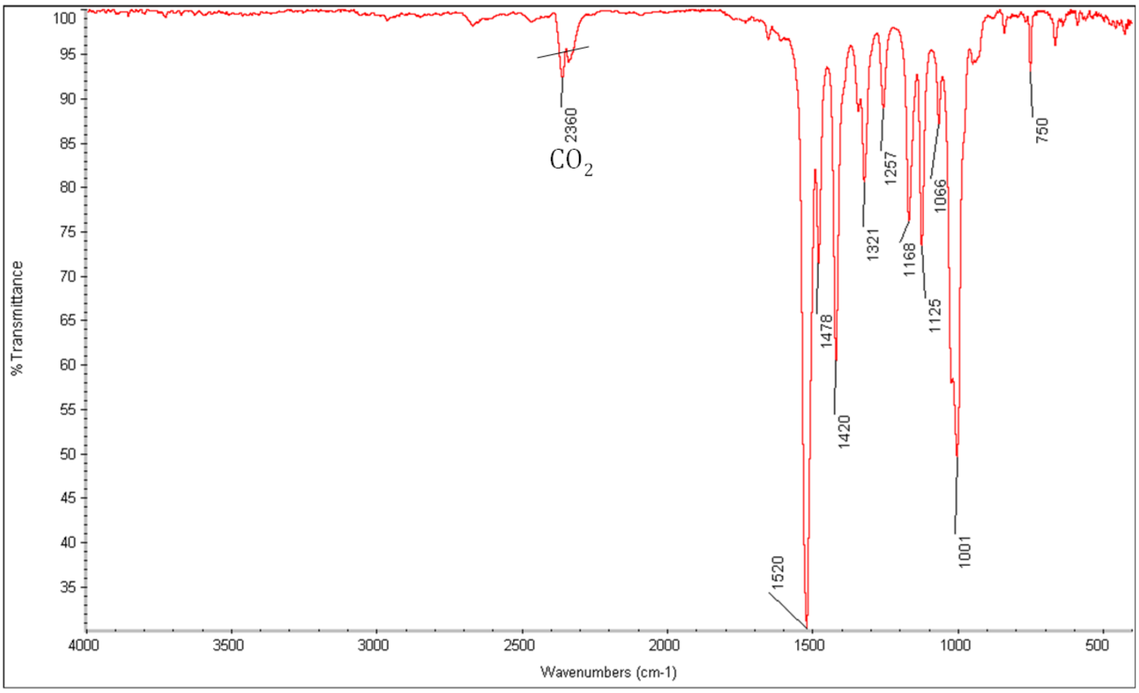

Figure S10. FT-IR spectrum of **CuPcF48**.

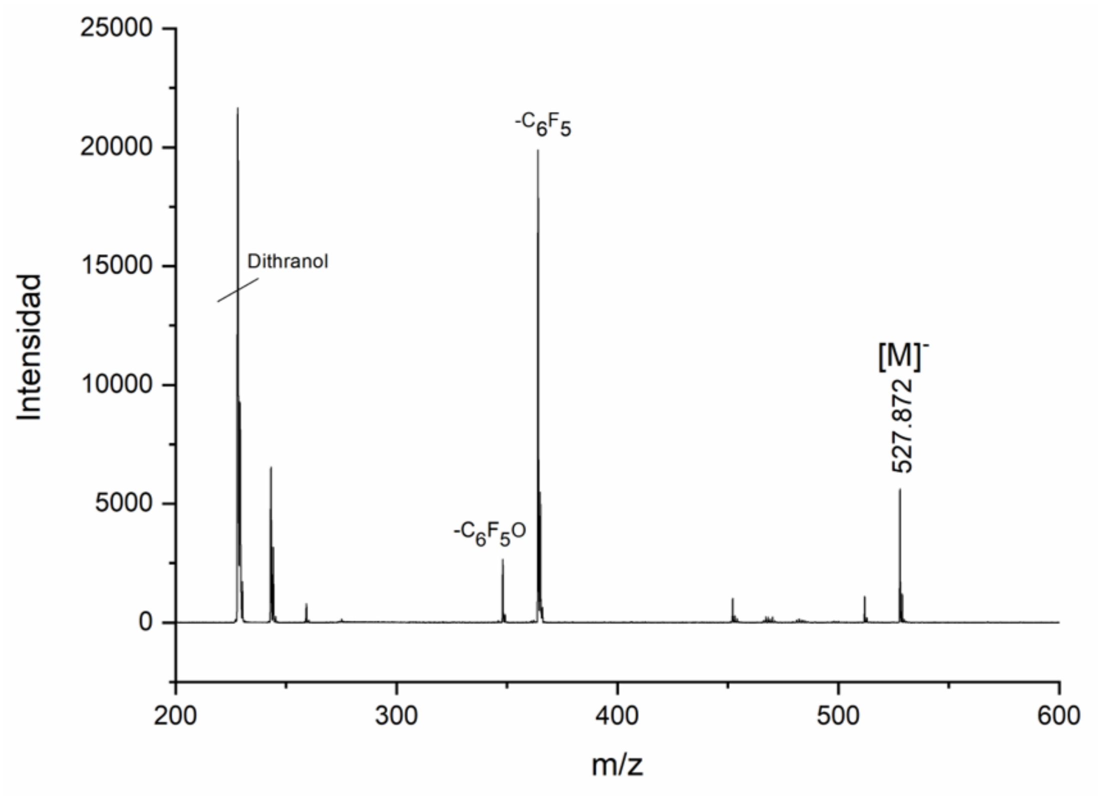

Figure S11. MS-MALDI-TOF of phthalonitrile **1**.

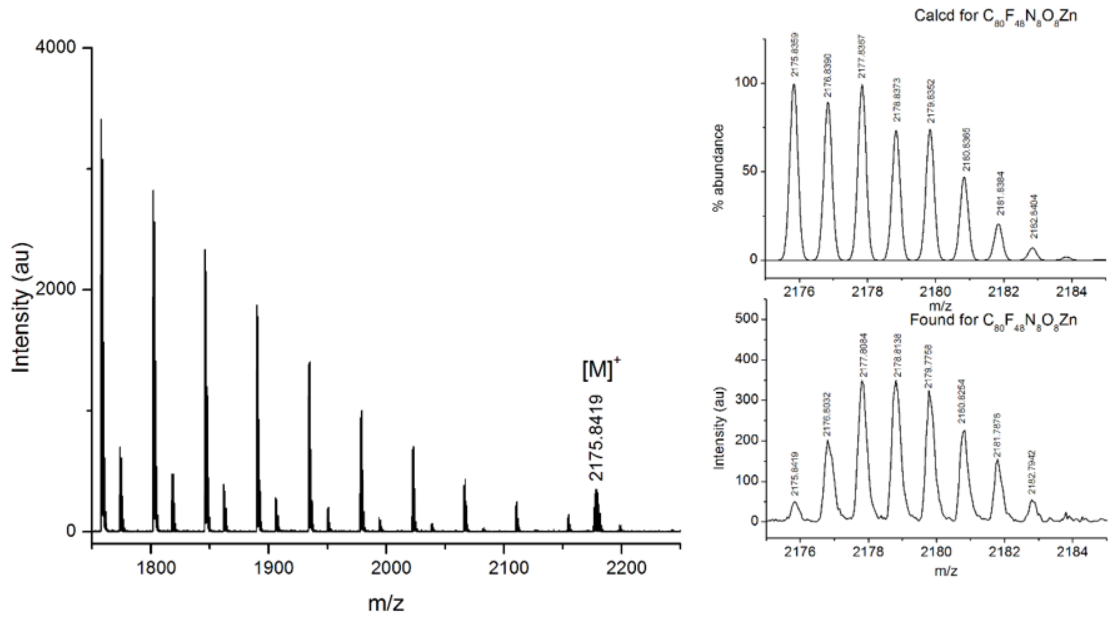

Figure S12. HR-MALDI-TOF of **ZnPcF$_{48}$**.

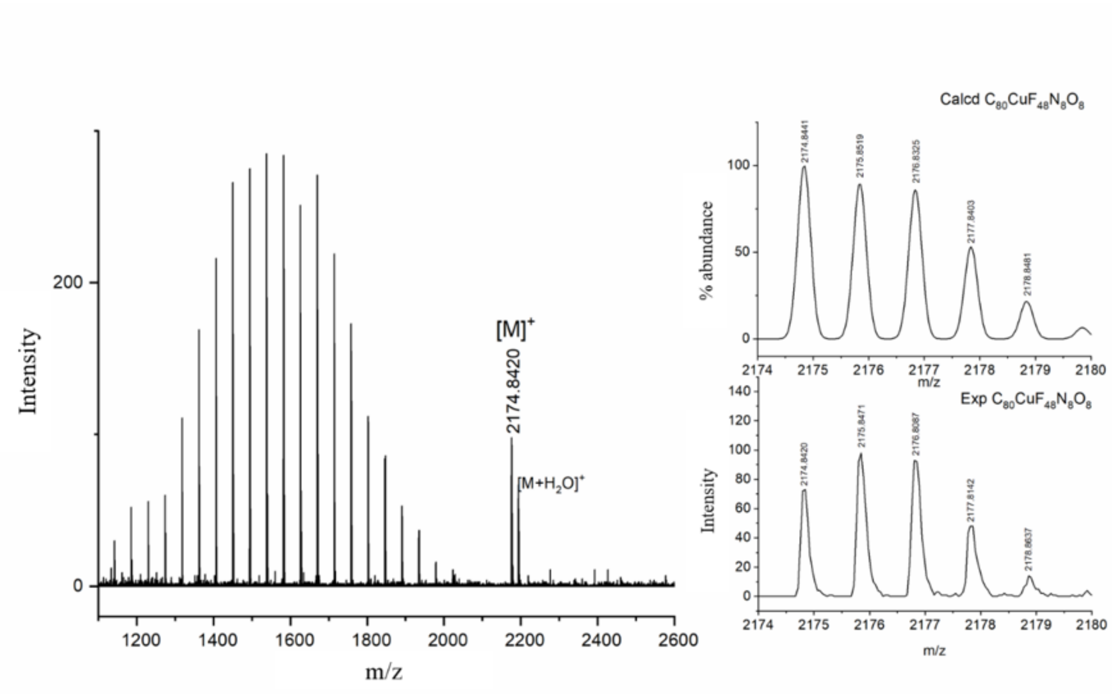

Figure S13. HR-MALDI-TOF of **CuPcF48**.

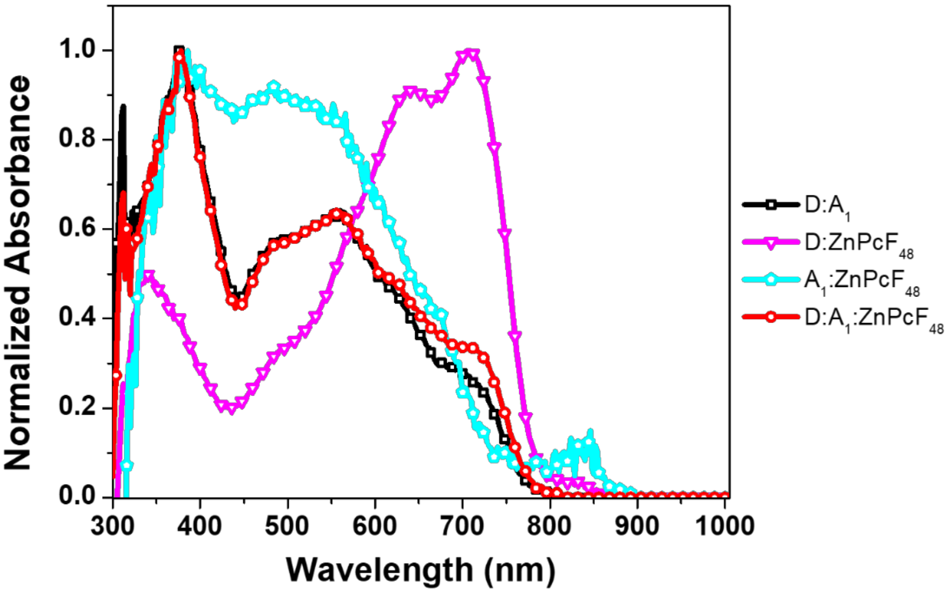

Figure S14. Normalized absorption spectra of reference blend, binary and ternary blend based on **ZnPcF48** in thin film.

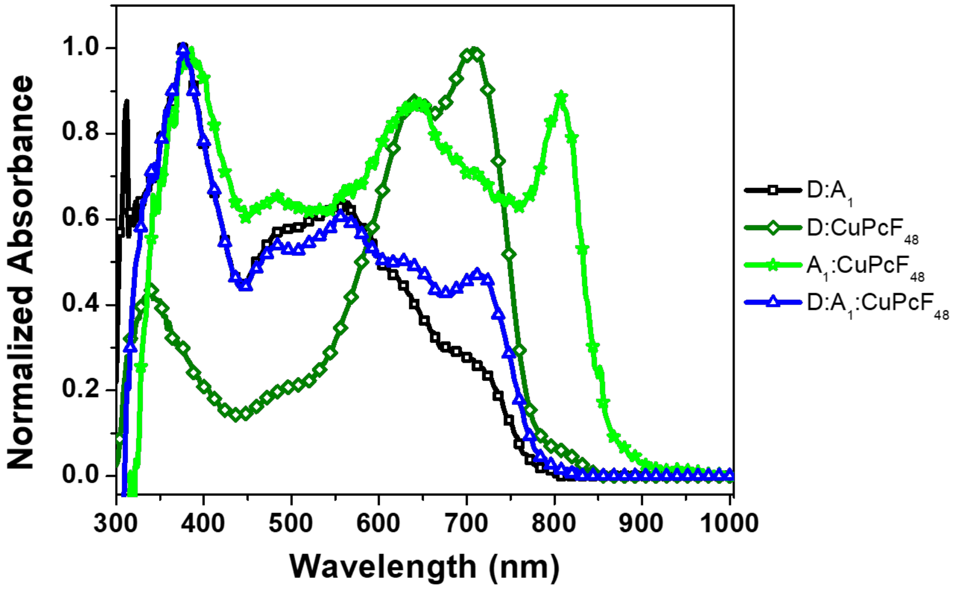

Figure S15. Normalized absorption spectra of reference blend, binary and ternary blend films based on **CuPcF48** in thin film.

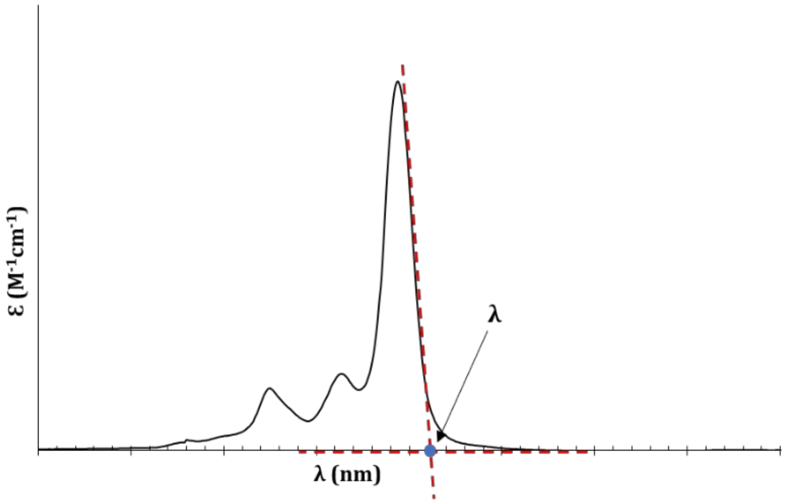

Figure S16. Schematic representation of a UV-Vis spectrum and the respective band gap energy estimation.

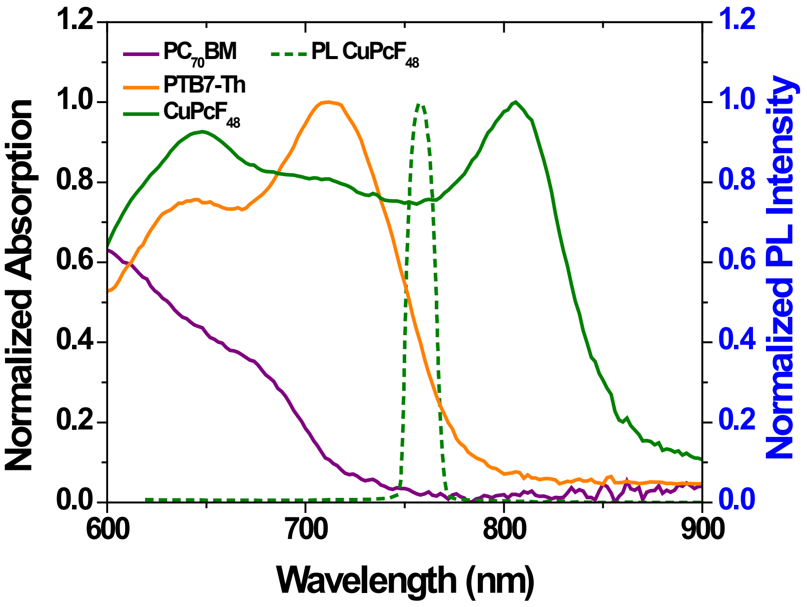

Figure S17. Normalized absorption spectra (left black y-axis, solid lines) of $PC_{70}BM$, PTB7-Th and **$CuPcF_{48}$** individual thin films, and photoluminescence spectra (right blue y-axis, dash line) of **$CuPcF_{48}$**. The excited wavelength for PL spectra is at 610 nm.

Table S1. Optimization of $D:A_1:A_2$ weight ratio and solvent vapour annealing (SVA) treatment for PTB7-Th:$PC_{70}BM$:**$CuPcF_{48}$** in inverted solar cells. Average values were obtained from over 10 devices for weight ratio optimization and over 3 devices for SVA treatment.

$D:A_1:A_2$ [%w/w]	SVA	V_{OC} [mA·cm^{-2}]	J_{SC} [V]	FF [%]	PCE [%]
1:1.5:0	-	16.82	0.78	68.22	9.01
	SVA	14.77	0.80	65.47	7.78
1:1.47:0.03	-	17.90	0.78	68.54	9.57
	SVA	16.18	0.79	64.86	8.37
1:1.44:0.06	-	14.08	0.77	71.18	7.69
	SVA	14.32	0.78	63.81	7.10
1:1.4:0.1	-	15.63	0.73	64.70	7.37
1:1.38:0.12	-	13.24	0.76	58.07	5.81
	SVA	11.79	0.33	28.30	1.12
1:0:1	-	0.06	0.32	0.24	0.01

Table S2. Optimization of electron transport layer for PTB7-Th:$PC_{70}BM$:**$ZnPcF_{48}$** in inverted solar cells. Average values were obtained from over 3 devices.

$D:A_1:A_2$ [%w/w]	ETL	V_{OC} [mA·cm^{-2}]	J_{SC} [V]	FF [%]	PCE [%]
1:1.5:0	PFN	16.82	0.78	68.22	9.01
	TiOx	10.76	0.78	71.18	5.92
1:1.4:0.1	PFN	14.25	0.78	64.47	7.12
	TiOx	11.87	0.70	58.84	4.88

Table S3. Optimization of D:A_1:A_2 weight ratio for PTB7-Th:$PC_{70}BM$:**ZnPcF48** in inverted solar cells. Average values were obtained from over 10 devices.

D:A_1:A_2 [%w/w]	V_{OC} [mA·cm^{-2}]	J_{SC} [V]	FF [%]	PCE [%]
1:1.5:0	16.82	0.78	68.22	9.01
1:1.47:0.03	14.25	0.78	64.47	7.12
1:1.4:0.1	14.25	0.78	64.47	7.12
1:0:1	0.29	0.33	0.50	0.03

The distribution of the third material within the bulk can be characterize experimentally by calculating its surface energy (γ) to further estimate its Flory-Huggins interaction parameter (χ) through contact angle measurement. The compatibility between two different materials can be estimated by the equation as follow:[1]

$$\gamma_{\mathrm{solvent}}(1+\cos\theta_{\mathrm{solvent}}) = \frac{4\,\gamma^{\mathrm{d}}_{\mathrm{solvent}}\gamma^{\mathrm{d}}}{\gamma^{\mathrm{d}}_{\mathrm{solvent}}+\gamma^{\mathrm{d}}} + \frac{\gamma^{\mathrm{p}}_{\mathrm{solvent}}\gamma^{\mathrm{p}}}{\gamma^{\mathrm{p}}_{\mathrm{solvent}}+\gamma^{\mathrm{p}}}$$

where $\gamma_{\mathrm{solvent}}$ are the surface tension of the solvent used, in this case is water or ethylene glycol. The superscript d and p represent the dispersion and polar components calculated using the contact angle with water or ethylene glycol.

Meanwhile, the Flory-Huggins interaction parameter can be calculated by the equation below:

$$\chi \propto \left(\sqrt{\gamma_{\mathrm{D}}} - \sqrt{\gamma_{\mathrm{A}}}\right)^2$$

Where γ_D and γ_A are the surface energy of the donor and acceptor material, respectively.

Table S4. Key parameters of contact angle measurements using water and ethylene glycol droplets.

Samples	θ_{water} (°)	θ_{EG} (°)	γ^p (mN m^{-1})	γ^d (mN m^{-1})	Surface tension (mN m^{-1})	χ with D	χ with A1
PTB7-Th	91.59	72.29	22.49	6.56	29.06	-	0.01
$PC_{70}BM$	90.60	65.54	17.34	10.86	28.21	0.01	-
$ZnPcF_{48}$	93.84	74.49	13.64	9.70	23.34	0.38	0.28

It can be seen from the **Table S4** that the χ values for the PTB7-Th:**ZnPcF48** (0.38) is much higher than those of $PC_{70}BM$:**ZnPcF48** (0.28), suggesting much lower miscibility or weaker interaction between **ZnPcF48** and PTB7-Th while a better mixed-phase between $PC_{70}BM$ and **ZnPcF48** is found. On the other hand, the χ values for the binary reference PTB7-Th: $PC_{70}BM$

(0.01) is much lower than the $ZnPcF_{48}$-based blend, indicating better miscibility and well mixed-phase in its blend. This result is in good agreement with the morphology characterization and device performance parameter.

Table S5. Extracted devices parameters from *J-V* dark measurements.

$D:A_1:A_2$ [%w/w]	R_S [Ω·cm²]	R_{Sh} [Ω·cm²]	J_0 [A·cm⁻²]
$D:A_1$	3.85	1.15×10^6	2×10^{-11}
$D:A_1:ZnPcF_{48}$	2.60	1.46×10^3	3×10^{-8}
$D:A_1:CuPcF_{48}$	3.77	1.86×10^6	1×10^{-11}
$D:ZnPcF_{48}$	112.48	2.13×10^6	1×10^{-7}
$D:CuPcF_{48}$	30.71	6.82×10^5	1×10^{-11}

Table S6. The values G_{max}, η_{diss}, and, η_{coll} for binary OSC PTB7-Th:$PC_{70}BM$ and the ternary OSCs PTB7-Th:$PC_{70}BM$:**$ZnPcF_{48}$** and PTB7-Th:$PC_{70}BM$:**$CuPcF_{48}$**.

Device	G_{max} [m^{-3} s^{-1}]	η_{diss} [%]	η_{coll} [%]
$D:A_1$	1.11×10^{28}	97.23	83.90
$D:A_1:ZnPcF_{48}$	8.71×10^{27}	97.98	83.63
$D:A_1:CuPcF_{48}$	1.16×10^{28}	98.89	84.01

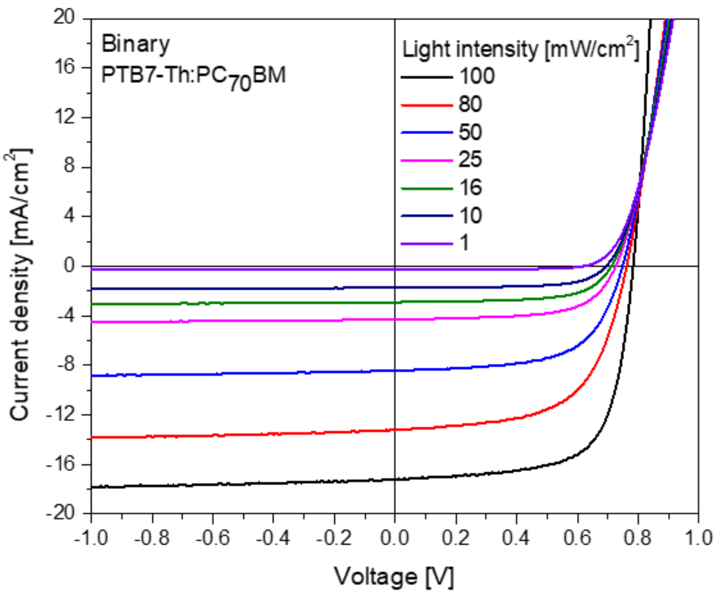

Figure S18. *J-V* characteristics of binary PTB7-Th:$PC_{70}BM$ at different light intensity, measured under AM 1.5G illumination using different optical filter.

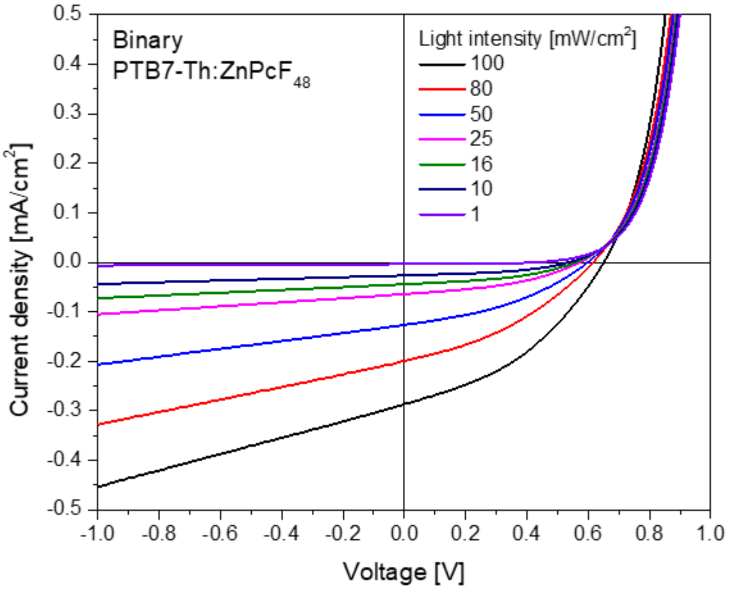

Figure S19. *J-V* characteristics of binary PTB7-Th:**ZnPcF48** at different light intensity, measured under AM 1.5G illumination using different optical filter.

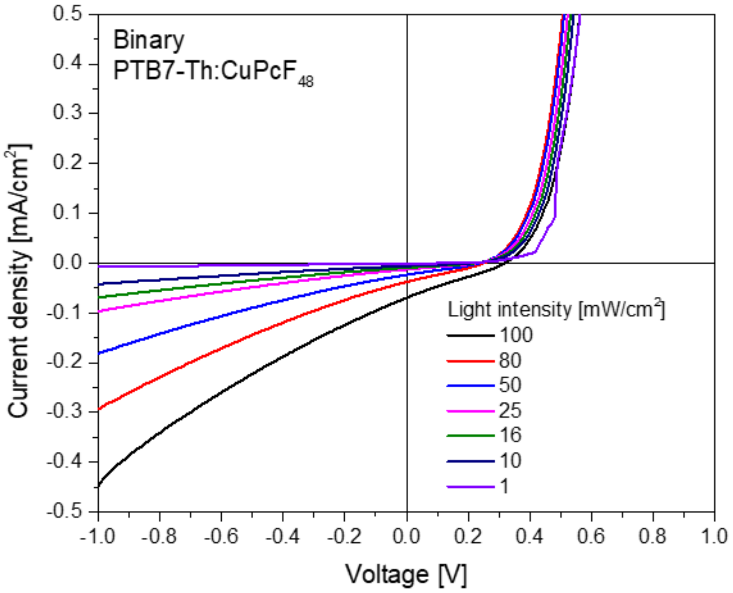

Figure S20. *J-V* characteristics of binary PTB7-Th:**CuPcF48** at different light intensity, measured under AM 1.5G illumination using different optical filter.

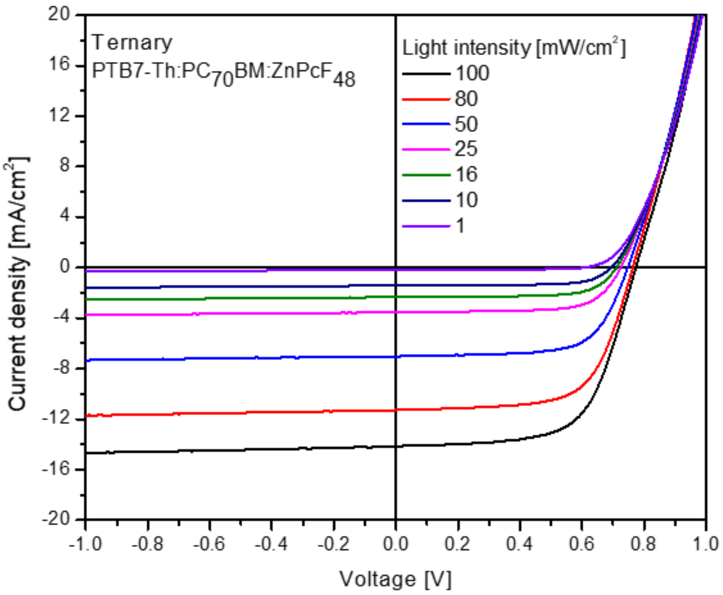

Figure S21. *J-V* characteristics of ternary PTB7-Th:$PC_{70}BM$:**$ZnPcF_{48}$** at different light intensity, measured under AM 1.5G illumination using different optical filter.

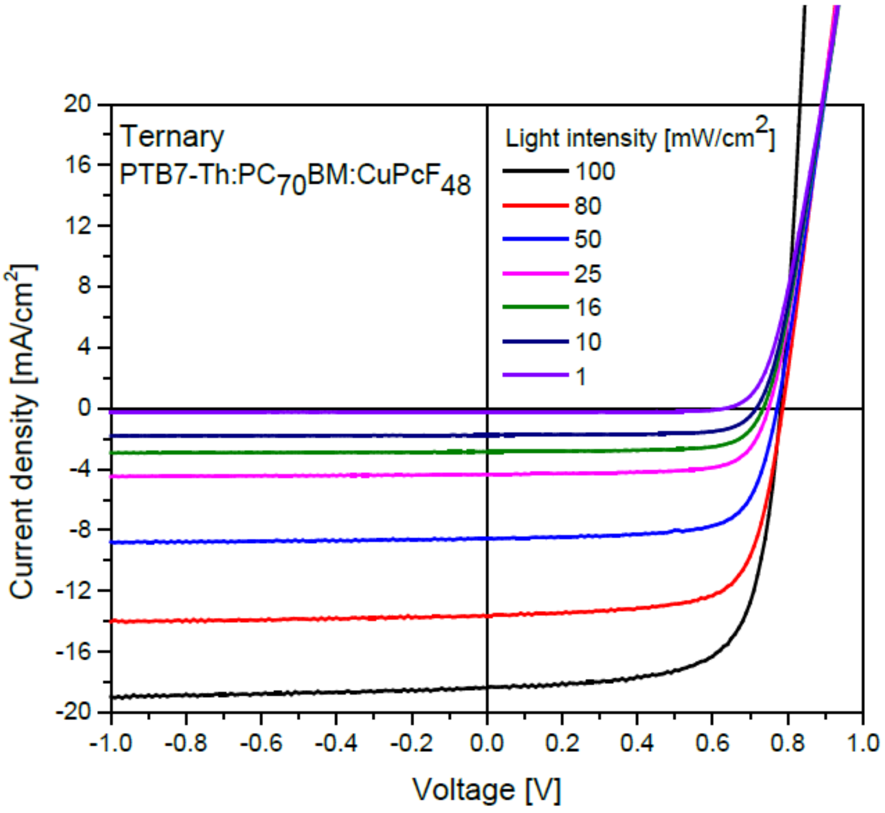

Figure S22. J-V characteristics of binary PTB7-Th:$PC_{70}BM$:**$CuPcF_{48}$** at different light intensity, measured under AM 1.5G illumination using different optical filter

REFERENCE

(1) Wu, S. Calculation of Interfacial Tension in Polymer Systems. *J. Polym. Sci. Part C Polym. Symp.* **1971**, *34* (1), 19–30. https://doi.org/10.1002/polc.5070340105.
